# Catalytic Isohypsic‐Redox Sequences for the Rapid Generation of C_sp3_‐Containing Heterocycles

**DOI:** 10.1002/chem.201804131

**Published:** 2018-11-02

**Authors:** Craig D. Smith, David Phillips, Alina Tirla, David J. France

**Affiliations:** ^1^ WestChem School of Chemistry University of Glasgow University Avenue Glasgow G12 8QQ UK

**Keywords:** heterocycles, homogeneous catalysis, isohypsic, palladium, tandem catalysis

## Abstract

Cross‐coupling reactions catalyzed by transition metals are among the most influential in modern synthetic chemistry. The vast majority of transition‐metal‐catalyzed cross‐couplings rely on a catalytic cycle involving alternating oxidation and reduction of the metal center and are generally limited to forging just one type of new bond per reaction (e.g., the biaryl linkage formed during a Suzuki cross‐coupling). This work presents an Isohypsic‐Redox Sequence (IRS) that uses one metal to effect two catalytic cycles, thereby generating multiple new types of bonds from a single catalyst source. We show that the IRS strategy is amenable to several widely used transformations including the Suzuki–Miyaura coupling, Buchwald–Hartwig amination, and Wacker oxidation. Furthermore, each of these reactions generates value‐added heterocycles with significant sp^3^‐C (3‐dimensional) content. Our results provide a general framework for generating complex products by using a single metal to fulfill multiple roles. By uniting different combinations of reactions in the isohypsic and redox phases of the process, this type of catalytic multiple bond‐forming platform has the potential for wide applicability in the efficient synthesis of functional organic molecules.

Transition metal catalysis is among the most common strategies in organic synthesis for the formation of C−C and C−X bonds.^1^ The vast majority of organometallic reactions rely on a single catalytic cycle to generate one new bond.^2^ Although the power of transition metal catalysis to effect previously unknown reactions has proved to be tremendously enabling, this “one reaction‐one bond” limitation fails to maximize the complexity of the products generated using these methods. Catalytic multiple‐bond‐forming strategies carry vast potential to impact the “economies of synthesis” through the rapid evolution of molecular complexity.^3^ We set out to develop a multiple‐bond‐forming reaction sequence that would use a single metal to effect multiple catalytic cycles by uniting an *isohypsic* reaction manifold with more common *redox* catalytic cycles.^4^

Most transition‐metal‐catalyzed reactions form a single new bond via a mechanistic cycle that involves alternating oxidative and reductive steps with respect to the metal catalyst. A smaller, but still widely used, set of metal‐catalyzed processes occurs without changes in the metal oxidation state, that is, in an *isohypsic* manifold. Common examples of such catalytic cycles include the conjugate addition of organoboronates to α,β‐unsaturated carbonyl compounds,^5^ Au^I^ or Co^III^‐catalyzed alkyne activations,^6^ metallocarbenoid reactions (e.g., Rh^II^‐catalyzed reactions of α‐diazocarbonyls),^7^ and the chain propagation phase of metal‐catalyzed alkene polymerization.^8^, ^9^

The preference for redox‐active catalytic cycles is maintained in the wide field of Pd‐catalyzed fine chemical synthesis. Most processes occur by some variant of the well‐known iterative sequence of oxidative addition, transmetallation, and reductive elimination. Nevertheless, many isohypsic Pd‐catalyzed processes are known, such as the addition of organoboronates to activated π‐bonds,^10^ cycloisomerization processes that terminate by protonation or β‐halide elimination,^11^ allylic rearrangements of esters or imidates,^12^ and the halo‐allylation of alkynes,^13^ among others.^14^

The mechanistic distinction between redox‐active and isohypsic catalysis carries an important consequence from a synthetic perspective, namely that functionality that is inert to the metal oxidation state present in the isohypsic process (such as the aryl halides typically involved in oxidative addition to Pd^0^) should be tolerated during an isohypsic reaction at a different oxidation state (e.g., Pd^II^). Subsequent alteration of the metal oxidation state (for example by the addition of a new reagent) allows for a second catalytic bond formation to occur using the same metal (Figure 1).

**Figure 1:**
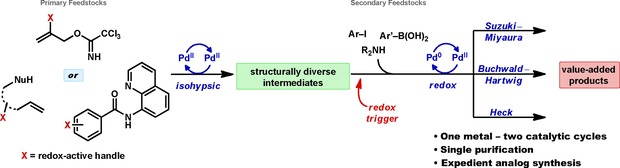
Overview of the isohypsic‐redox sequence (IRS) as an approach to complex molecule synthesis.

This type of transition from isohypsic to redox manifolds is an example of assisted tandem catalysis where one precatalyst effects two distinct catalytic processes using sequential reagent combinations to control the change in mechanism.^15^ Despite its potential for broad utility (based on the number of well elucidated catalytic cycles), this isohypsic‐redox strategy has seldom been used in the field of Pd‐catalysis, and never in the context of alkene difunctionalization.^16^

We have previously developed a Pd‐catalyzed alkene difunctionalization reaction that forms a heterocycle with concomitant creation of an sp^3^–sp^3^ C−C bond (Figure 2 b).^17^ This methodology was specifically designed to generate heterocycles with significant sp^3^‐C content, as studies of clinical success rates indicate a correlation between the progress of drug candidates through clinical trials and enhanced three‐dimensionality.^18^ An isotopic labeling study suggested the alkene heteroallylation process proceeds via an isohypsic mechanism involving a somewhat unusual β‐halide elimination step.^17^, ^19^ Here, we describe the development of an isohypsic‐redox sequence (IRS) based on the unification of alkene heteroallylation with transformative Pd‐catalyzed redox‐active processes such as the Suzuki–Miyaura coupling, Buchwald–Hartwig amination, and both the Wacker and Feringa–Grubbs aldehyde‐selective Wacker oxidation protocols (Figure 2 c).16d, 20 This IRS approach enhances molecular complexity by generating three new bonds in a single process while also forming a heterocycle and a new sp^3^–sp^3^ C−C bond.

**Figure 2:**
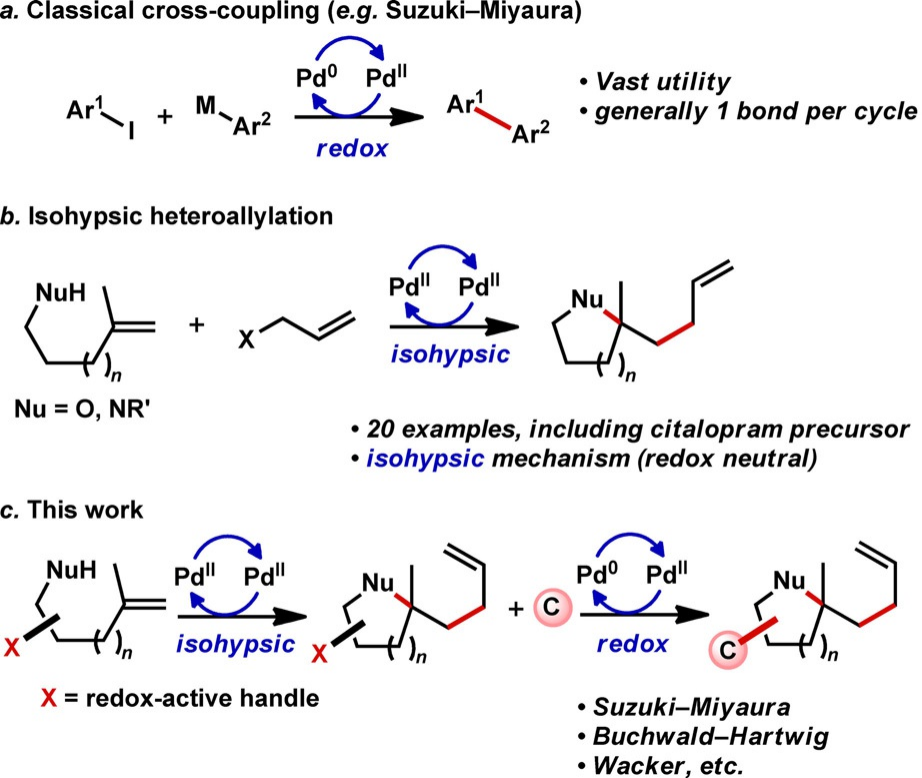
(a) Widely used cross‐coupling strategy. (b) Alkene heteroallylation reaction proceeding through isohypsic mechanism. (c) Postulated isohypsic‐redox tandem catalysis.

Our first task in achieving the planned IRS was to identify an appropriate substrate for the alkene heteroallylation process that contained a functional handle for use in a diverse array of subsequent redox reactions. As aryl halides are the most commonly used coupling partners in standard Pd‐catalyzed processes, bromophenol **1** was selected as our initial test case (Figure 3). Gratifyingly, this alkenyl phenol underwent the desired heteroallylation reaction to generate benzofuran **2** in good yield under our previously optimized conditions without engaging the aryl bromide, as expected by the all Pd^II^ catalytic cycle.^17^

**Figure 3:**
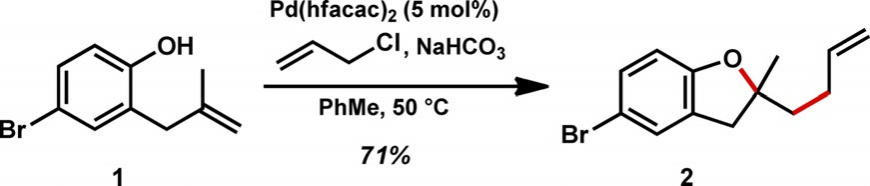
Heteroallylation of alkenyl phenol **1**. Isohypsic mechanism tolerates aryl bromide.

Once the heteroallylation in the presence of an aryl bromide had been demonstrated, we set out to establish our first IRS using the Suzuki–Miyaura cross‐coupling, the most common C−C bond‐forming reaction used by medicinal chemists.^21^ In this process, we were relying on the well‐precedented reduction of Pd^II^ to Pd^0^ by boronic acids to initiate the redox catalytic cycle.^22^ After optimization,^23^ including use of Buchwald dialkylbiaryl phosphine ligands,^24^ we were able to generate the desired biaryl coupling products in good yield through the two catalytic cycles (Figure 4). Substrate scoping studies demonstrated that both electron‐withdrawing and electron‐donating substituents were tolerated. By modifying the phosphine ligand to XPhos in the case of thiophene (**3 e**),^25^ and PPhos in the case of pyridine (**3 f**),^26^ we were able to effectively couple these heterocycles.

**Figure 4:**
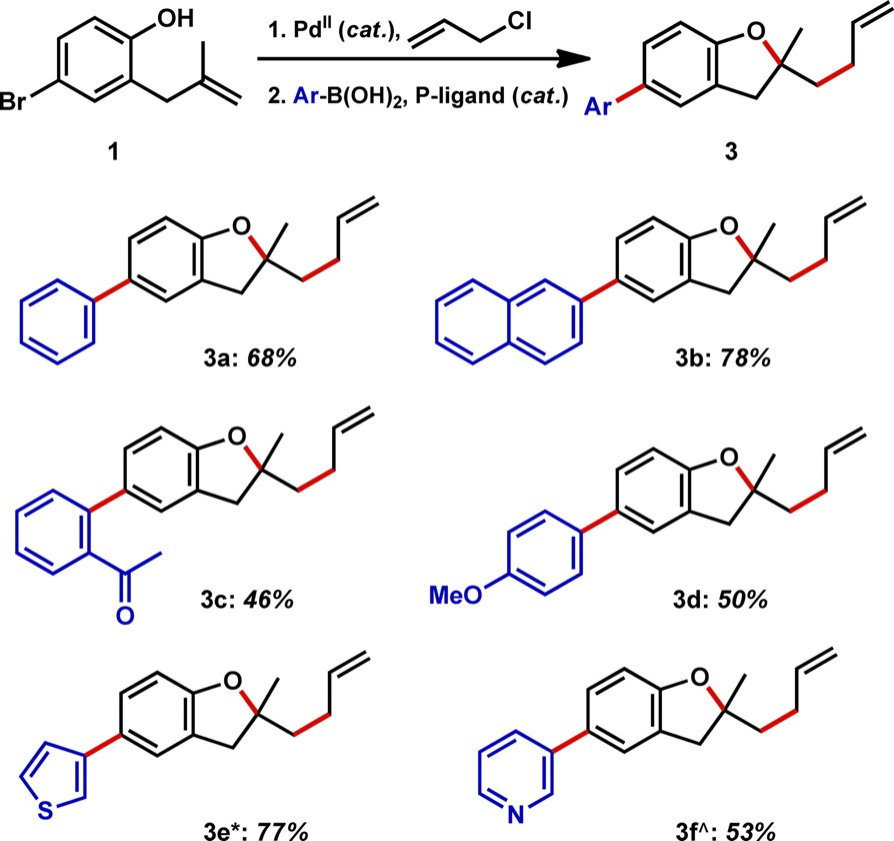
Tandem heteroallylation–Suzuki coupling. Isolated yields based on **1**. * SPhos replaced by XPhos, ^ SPhos replaced by PPhos.

Having demonstrated the capacity to form C−C bonds in the redox phase of the IRS process, we next chose to study C−N bond formation using the Buchwald–Hartwig amination.^27^ In this instance, reduction of the Pd^II^ was envisaged to occur via a β‐hydride elimination from a Pd^II^‐amine complex.^28^ Use of a dialkylbiaryl phosphine was again found to be advantageous in coupling with hexyl amine (Figure 5). In addition to primary amines, the coupling proceeded well with secondary amines to generate morpholine **4 c**, piperazine **4 d**, and aniline **4 e**.

**Figure 5:**
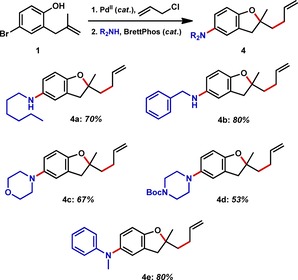
Tandem heteroallylation–Buchwald–Hartwig amination. Isolated yields based on **1**.

In order to extend the scope of the IRS platform beyond functionalized benzofurans, as well as to make use of the double bond that is installed by the isohypsic heteroallylation, we set out to combine the synthesis of N‐containing heterocycles with oxidation of the double bond as a redox step (Figure 6).^29^ The Pd^0^ generated at the end of the Wacker process would be re‐oxidized by an external oxidant to complete a redox cycle. After screening a range of conditions for the standard Wacker oxidation, such as varying the re‐oxidant system,^23^ we found that benzoquinone was the most effective (**5** to **6**). A methyl ketone was successfully installed in compounds containing both the isoquinolone and pyrrolopyrazinone ring systems. We then turned our attention to the possible aldehyde‐selective Wacker‐type alkene oxidation developed by Feringa and Grubbs et al.16d, 20 Using silver nitrite and copper(II) chloride as co‐catalysts resulted in formation of the expected aldehyde as the major product in a modest overall yield consistent with the yields reported for these two processes in isolation (**5** to **7**).20e Interestingly, the presence of a nitrile ligand (as used in earlier work by Feringa and Grubbs et al.) was found to be essential for the reaction to proceed.

**Figure 6:**
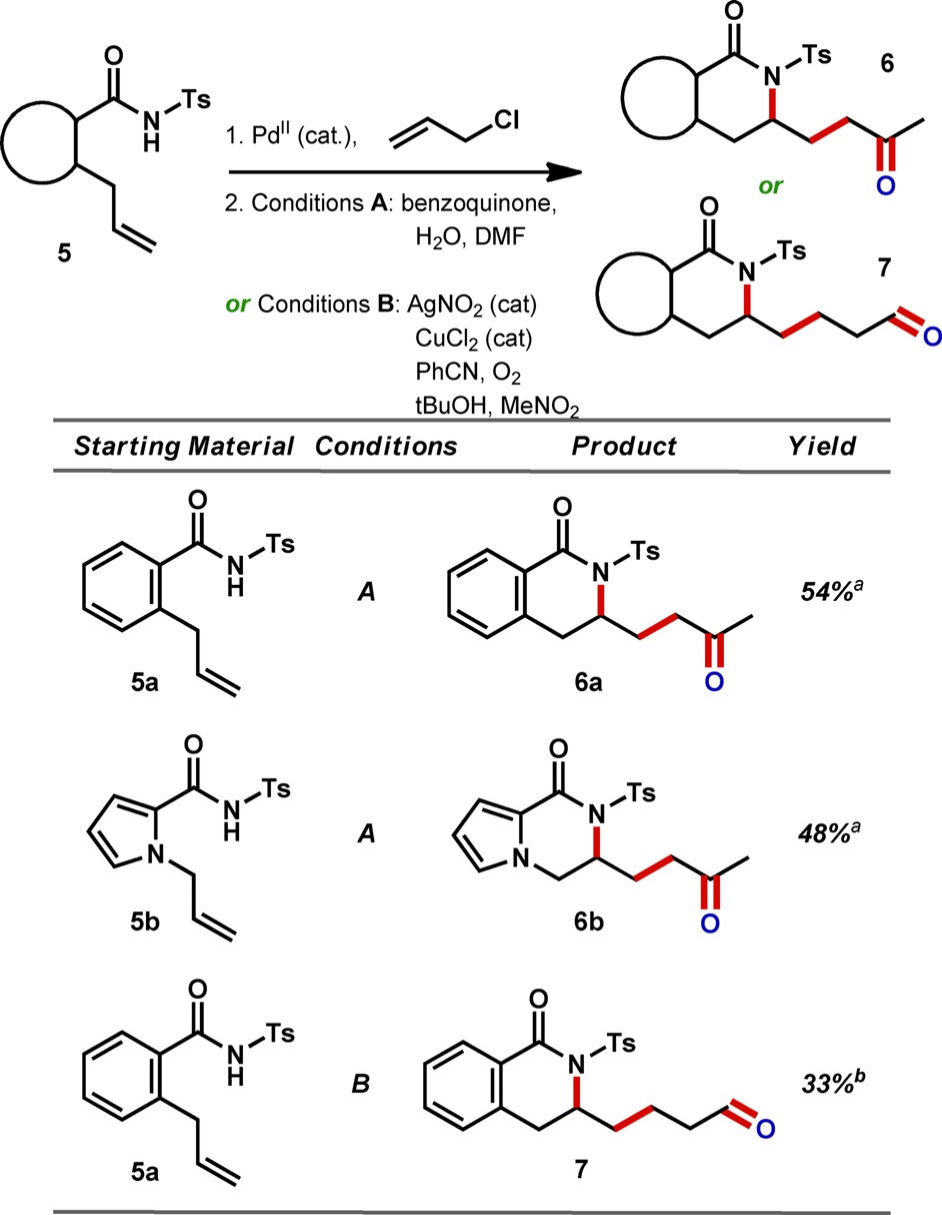
Tandem heteroallylation–Wacker‐type oxidations. Isolated yields based on **1**. (a) Isolated yield. (b) Yield determined by ^1^H NMR integration, additional 19 % yield of methyl ketone also observed. See the Supporting Information for details.

In summary, we have developed a suite of tandem catalytic processes based around the concept of linking the isohypsic (redox neutral) alkene heteroallylation reaction with well‐known redox catalytic cycles including the Suzuki–Miyaura, Buchwald–Hartwig, and Wacker transformations. In all cases, one metal is used to effect two different catalytic cycles, thereby providing a strategy for the rapid evolution of molecular complexity in the context of forming 3D heterocycles. Given the number of well‐elucidated catalytic cycles, expansion of the IRS concept has vast potential both within the field of Pd‐catalysis and beyond.

## Experimental Section

Representative procedure: A 4 mL screw‐top glass vial was charged with 4‐bromo‐2‐(2′‐methylallyl)phenol (**1**) (45.0 mg, 0.200 mmol), toluene (0.65 mL), allyl chloride (80.0 μL, 1.00 mmol), NaHCO_3_ (34.0 mg, 0.400 mmol) and Pd(hfacac)_2_ (5.00 mg, 0.0100 mmol) and the vial was sealed under ambient atmosphere. The resulting mixture was heated to 50 °C by immersion of the entire vial into a preheated aluminum block until the substrate had been consumed, as judged by TLC analysis. The reaction mixture was cooled to room temperature and the volatile components were evaporated in vacuo. To the vial was added toluene (0.4 mL), SPhos (8.00 mg, 0.0200 mmol), freshly ground K_3_PO_4_ (127 mg, 0.600 mmol) and phenylboronic acid (73.0 mg, 0.600 mmol) and the vial was sealed under ambient atmosphere. The mixture was then heated at 50 °C for 16 h. The reaction mixture was cooled to room temperature then purified directly by flash chromatography on silica gel (petroleum ether, then petroleum ether/EtOAc; 98:2) to give **3 a** (36 mg, 68 %).

## Conflict of interest

The authors declare no conflict of interest.

## Supporting information

As a service to our authors and readers, this journal provides supporting information supplied by the authors. Such materials are peer reviewed and may be re‐organized for online delivery, but are not copy‐edited or typeset. Technical support issues arising from supporting information (other than missing files) should be addressed to the authors.

Supplementary
