# Supplementary material for: Catalytic Isohypsic‐Redox Sequences for the Rapid Generation of Csp3‐Containing Heterocycles
Source: Chemistry. 2018 Nov 2;24(65):17201–4. doi: 10.1002/chem.201804131 (PMC6391974; doi:10.1002/chem.201804131)
Supplement: Supplementary file 1 — Supplementary [file CHEM-24-17201-s001.pdf]

CHEMISTRY
A European Journal

Supporting Information

Catalytic Isohypsic-Redox Sequences for the Rapid Generation of C_{sp3}-Containing Heterocycles

Craig D. Smith, David Phillips, Alina Tirla, and David J. France*[a]

chem_201804131_sm_miscellaneous_information.pdf

General Experimental Information

Reactions involving air-sensitive reagents and dry solvents were performed in glassware that had been dried in an oven (150 °C) or flame-dried prior to use. These reactions were carried out with the exclusion of air using an argon atmosphere. All microwave reactions were carried out using a Biotage Initiator system. NMR spectra were recorded on a Bruker DPX-400 spectrometer (^{1}H NMR at 400 MHz and ^{13}C NMR at 100 MHz) or a Bruker DPX-500 spectrometer (^{1}H NMR at 500 MHz and ^{13}C NMR at 125 MHz). Chemical shifts are reported in ppm. ^{1}H NMR spectra were recorded with $CDCl_3$ as the solvent using residual $CHCl_3$ (δ = 7.27) as internal standard or C_6D_6 as the solvent using residual C_6D_5H (δ = 7.16), and for ^{13}C NMR spectra the chemical shifts are reported relative to the central resonance of $CDCl_3$ (δ = 77.00) or C_6D_6 (δ = 128.39). Signals in NMR spectra are described as singlet (s), doublet (d), triplet (t), quartet (q), quintet (quint), septet (sept), multiplet (m), broad (br) or combination of these, which refers to the spin−spin coupling pattern observed. Spin−spin coupling constants reported are uncorrected. Two-dimensional (COSY, HSQC, HMBC, NOESY) NMR spectroscopy was used where appropriate to assist the assignment of signals in the ^{1}H and ^{13}C NMR spectra. IR spectra were obtained employing a Shimadzu FTIR-8400 instrument with a Golden Gate™ attachment that uses a type IIa diamond as a single reflection element so that the IR spectrum of the compound (solid or liquid) could be detected directly (thin layer). High resolution mass spectra were recorded under FAB, ESI or CI conditions by the analytical services at the University of Glasgow. Flash column chromatography was performed using forced flow of the indicated solvent system on EMD Geduran® Silica Gel 60 as solid support and HPLC graded solvents as eluent. Reactions were monitored by thin layer chromatography (TLC) on Merck silica gel 60 covered aluminium sheets. TLC plates were developed under UV-light and/or with an acidic ethanolicanisaldehyde solution or a $KMnO_4$-solution. Liquid reagents were distilled prior to use where stated. All reagents were purchased from commercial suppliers and used without further purification unless otherwise stated.

Experimental Details

4-bromo-2-(2'-methylallyl)phenol (1)

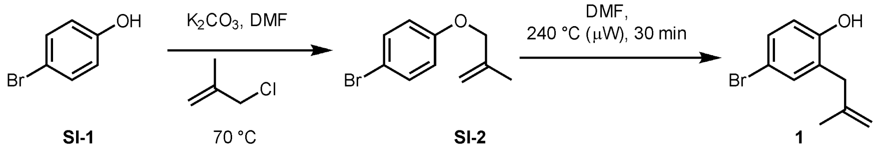

To a stirred suspension of K_2CO_3 (3.98 g, 28.8 mmol) in DMF (60 mL) was added 4-bromophenol (**SI-1**) (2.00 g, 11.6 mmol) followed by 3-chloro-2-methyl-1-propene (1.36 mL,

13.9 mmol). The resulting mixture was heated at 70 °C for 18 h then cooled to room temperature, quenched with water (25 mL) and extracted with Et_2O (2 x 25 mL). The combined organic extracts were washed with brine (2 x 25 mL), dried ($MgSO_4$), filtered and concentrated *in vacuo* to afford 1-(2'-methylallyloxy)-4-bromobenzene (**SI-2**) as a colourless oil (2.63 g, quant.) which was used without any further purification. Data corresponded to literature values.[1]

A solution of 1-(2'-methylallyloxy)-4-bromobenzene (**SI-2**) (2.00 g, 8.81 mmol) in DMF (5.9 mL) under argon was subjected to microwave irradiation at 240 °C for 30 minutes. The resulting mixture was diluted with water (25 mL), extracted with EtOAc (3 x 25 mL) and the combined organic extracts washed with brine (3 x 25 mL), dried ($MgSO_4$), filtered and concentrated *in vacuo*. Purification by flash chromatography (petroleum ether/EtOAc, 9:1) afforded the title compound (**1**) as a colourless oil (1.43 g, 72%). Data corresponded to literature values.[1]

2-(but-3'-en-1'-yl)-2-methyl-5-phenyl-2,3-dihydrobenzofuran (3a)

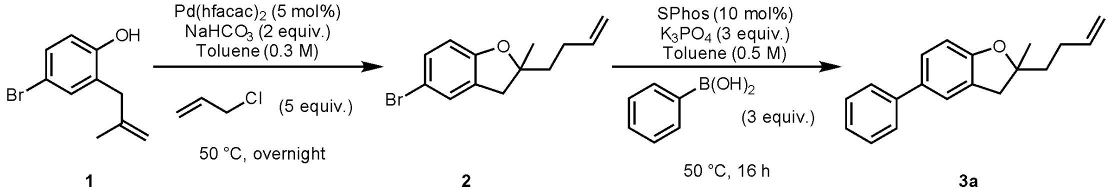

A 4 mL screw-top glass vial was charged with 4-bromo-2-(2'-methylallyl)phenol (**1**)(45.0 mg, 0.200 mmol), toluene (0.65 mL), allyl chloride (80.0 µL, 1.00 mmol), $NaHCO_3$ (34.0 mg, 0.400 mmol) and Pd(hfacac)$_2$ (5.00 mg, 0.0100 mmol) and the vial was sealed under ambient atmosphere. The resulting mixture was heated to 50 °C by immersion of the entire vial into a preheated aluminium block until the substrate had been consumed, as judged by TLC analysis. The reaction mixture was cooled to room temperature and the volatile components were evaporated *in vacuo*. To the vial was added toluene (0.4 mL), SPhos (8.00 mg, 0.0200 mmol), freshly ground K_3PO_4 (127 mg, 0.600 mmol) and phenylboronic acid (73.0 mg, 0.600 mmol) and the vial was sealed under ambient atmosphere. The mixture was then heated at 50 °C for 16 h. The reaction mixture was cooled to room temperature then purified directly by flash chromatography on silica gel (petroleum ether, then petroleum ether/EtOAc; 98:2) to give the title compound **3a** (36 mg, 68%).

^{1}H NMR(400 MHz, $CDCl_3$) δ 7.47–7.40 (2H, m), 7.35–7.24 (4H, m), 7.23–7.16 (1H, m), 6.71 (1H, d, *J* = 8.6 Hz), 5.91 (1H, ddt, *J* = 16.8, 10.1, 6.7), 4.96 (H, ddd, *J* = 17.1, 3.4, 1.7 Hz), 4.88 (1H, ddd, *J* = 10.2, 2.7, 1.7 Hz), 3.09 (1H, d, *J* = 15.6 Hz), 2.91 (1H, d, *J* = 15.6 Hz), 2.15–2.09 (2H, m), 1.81–1.75 (2H, m), 1.40 (3H, s); ^{13}C NMR (100 MHz, $CDCl_3$) δ 158.7,

141.5, 138.3, 133.6, 128.7 (x 2), 127.5, 127.1 (x 2), 126.7, 126.4, 123.9, 114.6, 109.5, 88.9, 41.3, 40.4, 28.5, 26.5; IR (thin film) 2976, 2360, 1479, 1265; HRMS (EI) exact mass calculated for $C_{19}H_{20}O$ $[M]^+$*m/z* 264.1514, found *m/z* 264.1519.

2-(but-3'-enyl)-2-methyl-5-(2-naphthyl)-2,3-dihydro-1-benzofuran (3b)

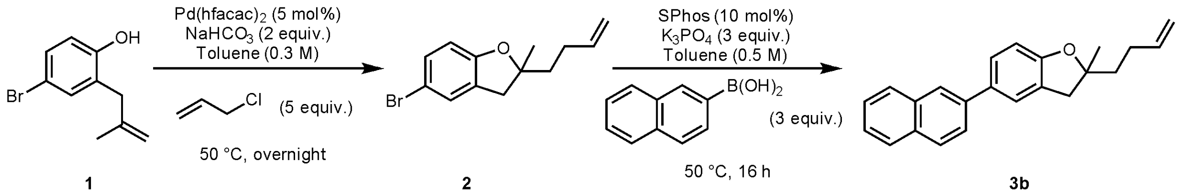

1 2 3b

A 4 mL screw-top glass vial was charged with 4-bromo-2-(2'-methylallyl)phenol (**1**)(45.0 mg, 0.200 mmol), toluene (0.65 mL), allyl chloride (80.0 µL, 1.00 mmol), $NaHCO_3$ (34.0 mg, 0.400 mmol) and $Pd(hfacac)_2$ (5.00 mg, 0.0100 mmol) and the vial was sealed under ambient atmosphere. The resulting mixture was heated to 50 °C by immersion of the entire vial into a preheated aluminium block until the substrate had been consumed, as judged by TLC analysis. The reaction mixture was cooled to room temperature and the volatile components were evaporated *in vacuo*. To the vial was added toluene (0.4 mL), SPhos (8.00 mg, 0.0200 mmol), freshly ground K_3PO_4 (127 mg, 0.600 mmol) and 2-naphthalene boronic acid (103 mg, 0.600 mmol) and the vial was sealed under ambient atmosphere. The mixture was then heated at 50 °C for 16 h. The reaction mixture was cooled to room temperature then purified directly by flash chromatography on silica gel (petroleum ether, then petroleum ether/EtOAc; 98:2) to give the title compound **3b** (49 mg, 78%).

^{1}H NMR (400 MHz, $CDCl_3$) δ 7.99 (1H, s), 7.88 (3H, m), 7.72 (1H, d, *J* = 8.6 Hz), 7.55–7.45 (4H, m), 6.71 (1H, d, *J* = 8.2 Hz), 5.94–5.84 (1H, m), 5.08 (1H, ddd, *J* = 17.1, 1.8, 1.6 Hz), 5.01 (1H, ddd, *J* = 10.2, 1.8, 1.3 Hz), 3.22 (1H, d, *J* = 15.6 Hz), 3.03 (1H, d, *J* = 15.6 Hz), 2.25 (2H, m), 1.91 (2H, m), 1.52 (3H, s); ^{13}C NMR (100 MHz, $CDCl_3$) δ 158.8, 138.8, 138.3, 133.8, 133.4, 132.2, 128.2, 128.0, 127.7 , 127.6, 127.5, 126.2, 125.6, 125.5, 124.9, 124.2, 114.6, 109.5, 89.1, 41.3, 40.4, 28.5, 26.5; IR (thin film) 2972, 2360, 1491, 1261, 734; HRMS (ESI) exact mass calculated for $C_{23}H_{22}NaO$ $[M+Na]^+$*m/z* 337.1563, found *m/z* 337.1536.

1-(2-(2'-(but-3''-en-1''-yl)-2'-methyl-2',3'-dihydrobenzofuran-5'-yl)phenyl)ethanone (3c)

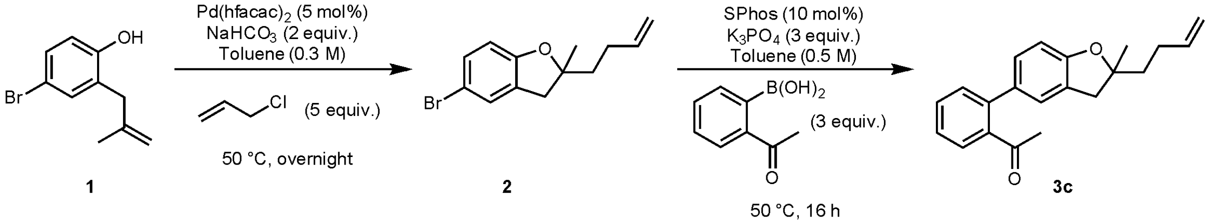

1 2 3c

A 4 mL screw-top glass vial was charged with 4-bromo-2-(2'-methylallyl)phenol (**1**)(45.0 mg,

0.200 mmol), toluene (0.65 mL), allyl chloride (80.0 µL, 1.00 mmol), $NaHCO_3$ (34.0 mg, 0.400 mmol) and $Pd(hfacac)_2$ (5.00 mg, 0.0100 mmol) and the vial was sealed under ambient atmosphere. The resulting mixture was heated to 50 °C by immersion of the entire vial into a preheated aluminium block until the substrate had been consumed, as judged by TLC analysis. The reaction mixture was cooled to room temperature and the volatile components were evaporated *in vacuo*. To the vial was added toluene (0.4 mL), SPhos (8.00 mg, 0.0200 mmol), freshly ground K_3PO_4 (127 mg, 0.600 mmol) and (2-acetylphenyl)boronic acid (98.0 mg, 0.600 mmol) and the vial was sealed under ambient atmosphere. The mixture was then heated at 50 °C for 16 h. The reaction mixture was cooled to room temperature then purified directly by flash chromatography on silica gel (petroleum ether, then petroleum ether/EtOAc; 98:2) to give the title compound **3c** (28 mg, 46%).

^{1}H NMR (400 MHz, $CDCl_3$) δ 7.53–7.46 (2H, m), 7.39–7.38 (1H, m), 7.37–7.35 (1H, m), 7.12–7.05 (2H, m), 6.78 (1H, d, J = 8.3 Hz), 5.85 (1H, ddt, J = 16.7, 10.3, 6.3), 5.05 (1H, ddd, J = 17.1, 3.4, 1.6 Hz), 4.98 (1H, ddd, J = 10.2, 3.0, 1.3 Hz), 3.14 (1H, d, J = 15.7 Hz), 2.99 (1H, d, J = 15.2 Hz), 2.25–2.15 (2H, m), 2.03 (3H, s), 1.90–1.84 (2H, m), 1.48 (3H, s); ^{13}C NMR (100 MHz, $CDCl_3$) δ 205.5, 159.1, 140.9, 140.6, 138.1, 132.5, 130.5, 130.1, 128.9, 127.7, 127.6, 126.8, 125.6, 114.7, 109.5, 89.2, 41.0, 40.3, 30.5, 28.4, 26.4; IR (thin film) 2914, 1681, 1275; HRMS (ESI) exact mass calculated for $C_{21}H_{22}O_2Na$ $[M+Na]^+$ *m/z* 329.1512, found *m/z* 329.1469.

2-(but-3'-enyl)-5-(4-methoxyphenyl)-2-methyl-2,3-dihydro-1-benzofuran (3d)

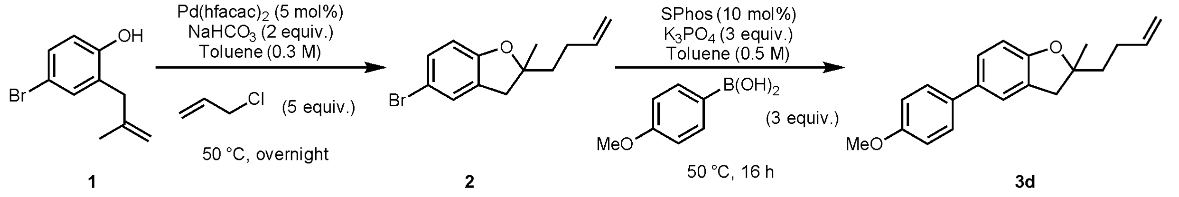

A 4 mL screw-top glass vial was charged with 4-bromo-2-(2'-methylallyl)phenol (**1**)(45.0 mg, 0.200 mmol), toluene (0.65 mL), allyl chloride (80.0 µL, 1.00 mmol), $NaHCO_3$ (34.0 mg, 0.400 mmol) and $Pd(hfacac)_2$ (5.00 mg, 0.0100 mmol) and the vial was sealed under ambient atmosphere. The resulting mixture was heated to 50 °C by immersion of the entire vial into a preheated aluminium block until the substrate had been consumed, as judged by TLC analysis. The reaction mixture was cooled to room temperature and the volatile components were evaporated *in vacuo*. To the vial was added toluene (0.4 mL), SPhos (8.00 mg, 0.0200 mmol), freshly ground K_3PO_4 (127 mg, 0.600 mmol) and 4-methoxy phenylboronic acid (92.0 mg, 0.600 mmol) and the vial was sealed under ambient atmosphere. The mixture was then heated at 50 °C for 16 h. The reaction mixture was

cooled to room temperature then purified directly by flash chromatography on silica gel (petroleum ether, then petroleum ether/EtOAc; 98:2) to give the title compound **3d** (30 mg, 50%).

^{1}H NMR (400 MHz, $CDCl_3$) δ 7.45 (2H, d, *J* = 8.9 Hz), 7.30 (2H, m), 6.95 (2H, d, *J* = 8.9 Hz), 6.78 (1H, d, *J* = 8.2 Hz), 5.87 (1H, ddt, *J* = 17.1, 10.3, 6.5 Hz), 5.05 (1H, ddd, *J* = 17.1, 1.8, 1.6 Hz), 4.97 (1H, ddd, *J* = 10.2, 1.8, 1.3 Hz), 3.85 (3H, s), 3.15 (1H, d, *J* = 15.6 Hz), 3.00 (1H, d, *J* = 15.6 Hz), 2.20 (2H, m), 1.86 (2H, m), 1.46 (3H, s). ^{13}C NMR (100 MHz, $CDCl_3$) δ 158.5, 158.2, 138.3 (x 2), 134.1, 133.2, 127.7 (x 2), 127.5, 126.7, 123.6, 114.6, 114.1, 109.5, 88.9, 55.3, 41.3, 40.4, 28.5, 26.5; IR (thin film) 2926, 1609, 1484, 1243, 1039, 814; HRMS (EI) exact mass calculated for $C_{20}H_{22}O_2$ $[M]^+$ *m/z* 294.1620, found *m/z* 294.1621.

2-(but-3'-enyl)-2-methyl-5-(3-thienyl)-2,3-dihydro-1-benzofuran (3e)

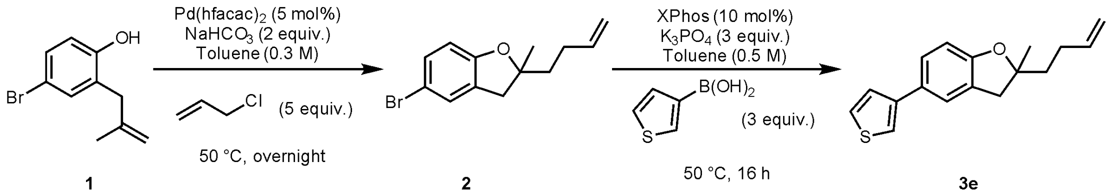

A 4 mL screw-top glass vial was charged with 4-bromo-2-(2'-methylallyl)phenol (**1**)(45.0 mg, 0.200 mmol), toluene (0.65 mL), allyl chloride (80.0 µL, 1.00 mmol), $NaHCO_3$ (34.0 mg, 0.400 mmol) and Pd(hfacac)$_2$ (5.00 mg, 0.0100 mmol) and the vial was sealed under ambient atmosphere. The resulting mixture was heated to 50 °C by immersion of the entire vial into a preheated aluminium block until the substrate had been consumed, as judged by TLC analysis. The reaction mixture was cooled to room temperature and the volatile components were evaporated *in vacuo*. To the vial was added toluene (0.4 mL), XPhos (10.0 mg, 0.0200 mmol), freshly ground K_3PO_4 (127 mg, 0.600 mmol) and 3-thienylboronic acid (77.0 mg, 0.600 mmol) and the vial was sealed under ambient atmosphere. The mixture was then heated at 50 °C for 16 h. The reaction mixture was cooled to room temperature then purified directly by flash chromatography on silica gel (petroleum ether, then petroleum ether/EtOAc; 98:2) to give the title compound **3e** (42 mg, 77%).

^{1}H NMR (400 MHz, $CDCl_3$) δ 7.36 (5H, m), 6.78 (1H, d, *J* = 8.4 Hz), 5.87 (1H, ddt, *J* = 17.1, 10.3, 6.5 Hz), 5.06 (1H, ddd, *J* = 17.1, 1.8, 1.6 Hz), 4.99 (1H, ddd, *J* = 10.1, 1.8, 1.3 Hz), 3.15 (1H, d, *J* = 15.6 Hz), 3.00 (1H, d, *J* = 15.6 Hz), 2.21 (2H, m), 1.87 (2H, m), 1.49 (3H, s). ^{13}C NMR (100 MHz, $CDCl_3$) δ 158.8, 142.6, 138.3, 128.4, 127.6, 126.5, 126.4, 125.9, 123.4, 118.5, 114.6, 109.5, 89.1, 41.3, 40.4, 28.5, 26.5; IR (thin film) 3104, 2972, 2360, 1483, 1256, 773; HRMS (EI) exact mass calculated for $C_{17}H_{18}OS$ $[M]^+$ *m/z* 270.1078, found *m/z* 270.1076.

3-(2'-(but-3''-en-1''-yl)-2'-methyl-2',3'-dihydrobenzofuran-5'-yl)pyridine (3f)

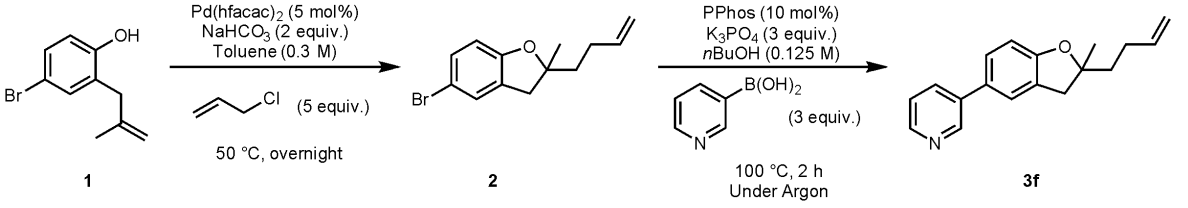

A 4 mL screw-top glass vial was charged with 4-bromo-2-(2'-methylallyl)phenol (**1**)(57.0 mg, 0.250 mmol), toluene (0.83 mL), allyl chloride (100 µL, 1.25 mmol), $NaHCO_3$ (42.0 mg, 0.500 mmol) and Pd(hfacac)$_2$ (7.0 mg, 0.0125 mmol) and the vial was sealed under ambient atmosphere. The resulting mixture was heated to 50 °C by immersion of the entire vial into a preheated aluminium block until the substrate had been consumed, as judged by TLC analysis. The reaction mixture was cooled to room temperature and the volatile components were evaporated *in vacuo*. Following a modified literature procedure,[2] to the vial was added PPhos (16.0 mg, 0.0250 mmol), *n*BuOH (1 mL) and argon was bubbled through the mixture. The reaction was allowed to stir for 5 minutes at room temperature. Freshly ground K_3PO_4 (159 mg, 0.750 mmol), 3-pyridinylboronic acid (92.0 mg, 0.750 mmol) and *n*BuOH (1 mL) were added to the reaction mixture and the vial was sealed under an argon atmosphere. The mixture was then heated to 100 °C for 2 h using the aluminium block. The reaction was allowed to cool to room temperature and quenched with water and the organic layers were extracted with EtOAc (3 x 20 mL). The combined organic extracts were dried over $MgSO_4$, and concentrated *in vacuo*. The crude mixture was purified by flash chromatography on silica gel (petroleum ether/EtOAc; 8:2) to give the title compound **3f** (35mg, 53%).

^{1}H NMR (400 MHz, $CDCl_3$) δ 8.81 (1H, br. s), 8.54 (1H, br. s), 7.84−7.76 (1H, m), 7.39−7.29 (3H, m), 6.83 (1H, d, *J* = 8.2 Hz), 5.85 (1H, ddt, *J* = 16.8, 10.3, 6.4), 5.05 (1H, ddd, *J* = 17.1, 3.4, 1.5 Hz), 4.97 (1H, ddd, *J* = 10.3, 2.9, 1.3 Hz), 3.17 (1H, d, *J* = 15.7 Hz), 3.01 (1H, d, *J* = 15.7 Hz), 2.24−2.16 (2H, m), 1.90−1.84 (2H, m), 1.49 (3H, s); ^{13}C NMR (100 MHz, $CDCl_3$) δ 159.3, 147.9, 147.5, 138.1, 133.8, 129.9, 128.0 (x 2), 127.3 (x 2), 123.9, 114.7, 109.9, 89.2, 41.1, 40.3, 28.4, 26.4; IR (thin film) 3074, 2928, 849, 71; HRMS (ESI) exact mass calculated for $C_{18}H_{19}NOH$ $[M+H]^+$ *m/z* 266.1539, found *m/z* 266.1532.

2-(but-3'-en-1'-yl)-*N*-hexyl-2-methyl-2,3-dihydrobenzofuran-5-amine (4a)

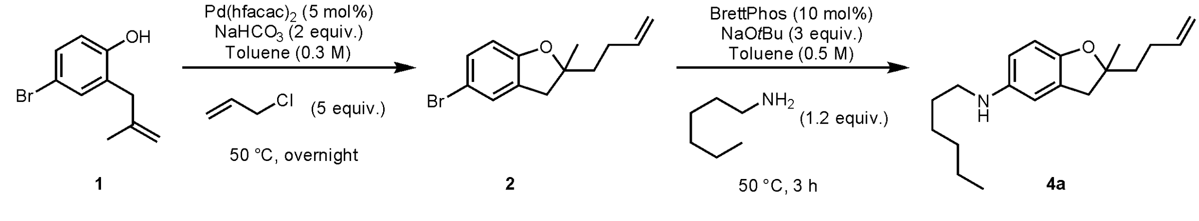

A 4 mL screw-top glass vial was charged with 4-bromo-2-(2'-methylallyl)phenol (**1**)(45 mg, 0.20 mmol), toluene (0.65 mL), allyl chloride (80 μL, 1.0 mmol), $NaHCO_3$ (34 mg, 0.40 mmol) and $Pd(hfacac)_2$ (5.0 mg, 0.010 mmol) and the vial was sealed under ambient atmosphere. The resulting mixture was heated to 50 °C by immersion of the entire vial into a preheated aluminium block until the substrate had been consumed, as judged by TLC analysis. The reaction mixture was cooled to room temperature and the volatile components were evaporated *in vacuo*. To the vial was added BrettPhos (10 mg, 0.020 mmol), NaO*t*Bu(58 mg, 0.60 mmol) and toluene (0.2 mL). The mixture was allowed to stir for 5 minutes at room temperature. Next, toluene (0.2 mL) and hexylamine (30 μL, 0.24 mmol) were added and the vial was sealed under ambient atmosphere. The mixture was then heated at 50 °C for 3 h. The reaction mixture was cooled to room temperature, quenched with water and the organic layers were extracted with Et_2O (3 x 20 mL). The combined organic extracts were then washed with brine, dried over $MgSO_4$ and concentrated *in vacuo.* The crude mixture was then purified by flash chromatography on silica gel (petroleum ether/EtOAc; 95:5) to give the title compound **4a** (40 mg, 70%).

^{1}H NMR (500 MHz, $CDCl_3$) δ 6.59 (1H, d, *J* = 8.3 Hz), 6.50 (1H, s), 6.44−6.39 (1H, m), 5.84 (1H, ddt, *J* = 16.6, 10.2, 6.4 Hz), 5.05 (1H, ddd, *J* = 17.1, 3.4, 1.6 Hz), 4.95 (1H, ddd, *J* = 10.0, 3.0, 1.1 Hz), 3.07−3.02 (3H, m), 2.88 (1H, d, *J* = 15.4 Hz), 2.21−2.13 (2H, m), 1.84−1.78 (2H, m), 1.64−1.56 (2H, m), 1.44−1.38 (5H, m), 1.35−1.30 (4H, m), 0.91 (3H, t, *J* = 6.9 Hz); ^{13}C NMR (125 MHz, $CDCl_3$) δ 152.1, 141.5, 138.4, 127.7, 114.4, 113.5, 111.3, 109.4, 87.8, 46.0, 41.6, 40.3, 31.6, 29.3, 28.4, 26.8, 26.4, 22.6, 14.0; IR (thin film) 2928, 1641, 910, 731; HRMS (ESI) exact mass calculated for $C_{19}H_{29}NOBrH$ $[M+H]^+$ *m/z* 288.2322, found *m/z* 288.2309.

***N*-benzyl-2-(but-3'-en-1'-yl)-2-methyl-2,3-dihydrobenzofuran-5-amine (4b)**

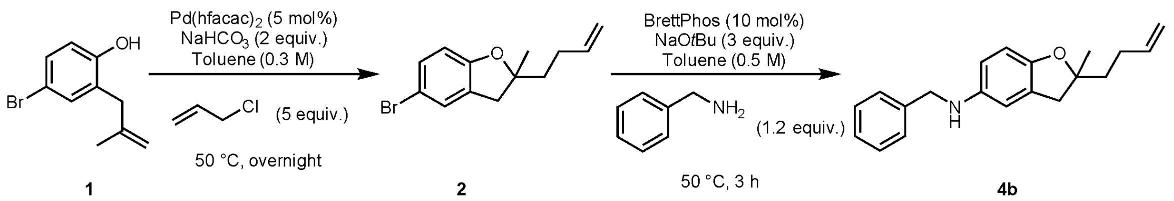

A 4 mL screw-top glass vial was charged with 4-bromo-2-(2'-methylallyl)phenol (**1**)(45 mg, 0.20 mmol), toluene (0.65 mL), allyl chloride (80 μL, 1.0 mmol), $NaHCO_3$ (34 mg, 0.40 mmol) and $Pd(hfacac)_2$ (5.0 mg, 0.010 mmol) and the vial was sealed under ambient atmosphere. The resulting mixture was heated to 50 °C by immersion of the entire vial into a preheated aluminium block until the substrate had been consumed, as judged by TLC analysis. The reaction mixture was cooled to room temperature and the volatile components were

evaporated *in vacuo*. To the vial was added BrettPhos (10 mg, 0.020 mmol), freshly ground NaO*t*Bu(58 mg, 0.60 mmol) and toluene (0.2 mL). The mixture was allowed to stir for 5 minutes at room temperature. Next, toluene (0.2 mL) and benzylamine (26 µL, 0.24 mmol) were added and the vial was sealed under ambient atmosphere. The mixture was then heated at 50 °C for 3 h. The reaction mixture was cooled to room temperature, quenched with water and the organic layers were extracted with Et_2O (3 x 20 mL). The combined organic extracts were then washed with brine, dried over $MgSO_4$ and concentrated *in vacuo*. The crude mixture was then purified by flash chromatography on silica gel (petroleum ether/EtOAc, 95:5) to give the title compound **4b** (47mg, 80%).

^{1}H NMR (500 MHz, $CDCl_3$) δ 7.41−7.34 (4H, m), 7.31−7.28 (1H, m), 6.59 (1H, d, *J* = 8.5 Hz), 6.54−6.52 (1H, m), 6.47−6.43 (1H, m), 5.85 (1H, ddt, *J* = 17.0, 10.3, 6.4 Hz), 5.03 (1H, ddd, *J* = 17.1, 3.3, 1.6 Hz), 4.96 (1H, ddd, *J* = 10.2, 3.1, 1.4 Hz), 4.27 (2H, s), 3.69 (1H, br. s), 3.04 (1H, d, *J* = 15.5 Hz), 2.88 (1H, d, *J* = 15.5 Hz), 2.21−2.14 (2H, m), 1.83−1.78 (2H, m), 1.42 (3H, s); ^{13}C NMR (125 MHz, $CDCl_3$) δ 151.7, 142.3, 139.8, 138.4, 128.6 (x 2), 127.7, 127.6 (x 2), 127.1, 114.4, 112.6, 110.6, 109.4, 87.7, 49.7, 41.7, 40.3, 28.4, 26.4; IR (thin film) 3066, 1492, 1222, 904; HRMS (ESI) exact mass calculated for $C_{20}H_{23}NH$ $[M+H]^+$*m/z* 294.1852, found *m/z* 294.1838.

4-(2'-(but-3''-en-1''-yl)-2'-methyl-2',3'-dihydrobenzofuran-5'-yl)morpholine (4c)

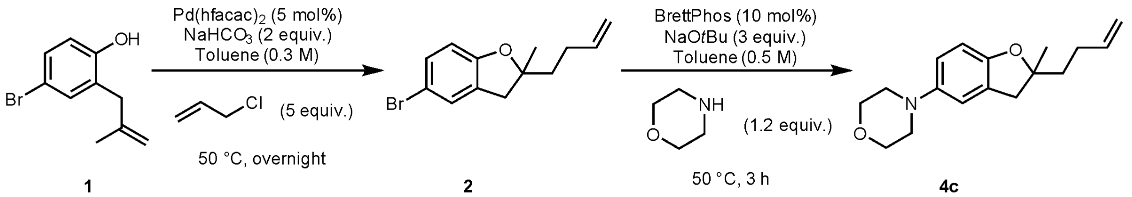

1 2 4c

A 4 mL screw-top glass vial was charged with 4-bromo-2-(2'-methylallyl)phenol (**1**)(45 mg, 0.20 mmol), toluene (0.65 mL), allyl chloride (80 µL, 1.0 mmol), $NaHCO_3$ (34 mg, 0.40 mmol) and $Pd(hfacac)_2$ (5.0 mg, 0.010 mmol) and the vial was sealed under ambient atmosphere. The resulting mixture was heated to 50 °C by immersion of the entire vial into a preheated aluminium block until the substrate had been consumed, as judged by TLC analysis. The reaction mixture was cooled to room temperature and the volatile components were evaporated *in vacuo*. To the vial was added BrettPhos (10 mg, 0.020 mmol), NaO*t*Bu(58 mg, 0.60 mmol) and toluene (0.2 mL). The mixture was allowed to stir for 5 minutes at room temperature. Next, toluene (0.2 mL) and morpholine (20 µL, 0.24 mmol) were added and the vial was sealed under ambient atmosphere. The mixture was then heated at 50 °C for 3 h. The reaction mixture was cooled to room temperature, quenched with water and the organic layers were extracted with Et_2O (3 x 20 mL). The combined organic extracts were then

washed with brine, dried over $MgSO_4$ and concentrated *in vacuo*. The crude mixture was then purified by flash chromatography on silica gel (petroleum ether/EtOAc, 85:15) to give the title compound **4c** (37 mg, 67%).

^{1}H NMR (400 MHz, $CDCl_3$) δ 6.83−6.78 (1H, m), 6.74−6.69 (1H, m), 6.66 (1H, d, J = 8.7 Hz), 5.84 (1H, ddt, J = 16.7, 10.0, 6.8 Hz), 5.07−4.99 (1H, m), 4.98−4.92 (1H, m), 3.89−3.82 (4H, m), 3.08 (1H, d, J = 15.7 Hz), 3.05−3.01 (4H, m), 2.92 (1H, d, J = 15.7 Hz), 2.21−2.12 (2H, m), 1.86−1.76 (2H, m), 1.43 (3H, s); ^{13}C NMR (100 MHz, $CDCl_3$) δ 153.8, 145.5, 138.3, 127.6, 116.9, 114.9, 114.5, 109.3, 88.3, 67.0 (x 2), 51.7 (x 2), 41.6, 40.3, 28.4, 26.4; IR (thin film) 2962, 1492, 1120, 883; HRMS (ESI) exact mass calculated for $C_{17}H_{23}NO_2H$ [M+H]$^+$ *m/z* 274.1802, found *m/z* 274.1792.

***tert*-butyl 4-(2'-(but-3''-en-1''-yl)-2'-methyl-2',3'-dihydrobenzofuran-5'-yl)piperazine-1-carboxylate (4d)**

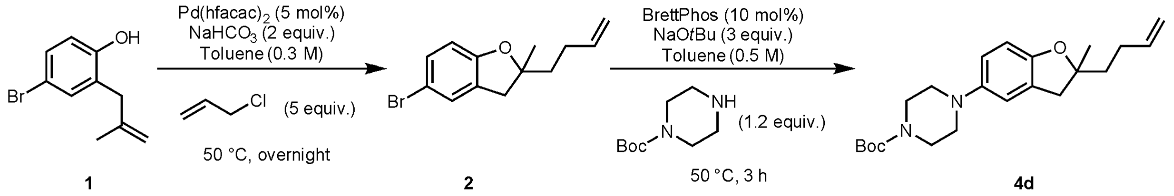

A 4 mL screw-top glass vial was charged with 4-bromo-2-(2'-methylallyl)phenol (**1**)(45 mg, 0.20 mmol), toluene (0.65 mL), allyl chloride (80 µL, 1.0 mmol), $NaHCO_3$ (34 mg, 0.40 mmol) and Pd(hfacac)$_2$ (5.0 mg, 0.010 mmol) and the vial was sealed under ambient atmosphere. The resulting mixture was heated to 50 °C by immersion of the entire vial into a preheated aluminium block until the substrate had been consumed, as judged by TLC analysis. The reaction mixture was cooled to room temperature and the volatile components were evaporated *in vacuo*. To the vial was added BrettPhos (10 mg, 0.020 mmol), NaO*t*Bu(58 mg, 0.60 mmol) and toluene (0.2 mL). The mixture was allowed to stir for 5 minutes at room temperature. Next, toluene (0.2 mL) and *tert*-butyl piperazine-1-carboxylate (40 mg, 0.24 mmol) were added and the vial was sealed under ambient atmosphere. The mixture was then heated at 50 °C for 3 h. The reaction mixture was cooled to room temperature, quenched with water and the organic layers were extracted with Et_2O (3 x 20 mL). The combined organic extracts were then washed with brine, dried over $MgSO_4$ and concentrated *in vacuo*. The crude mixture was then purified directly by flash chromatography on silica gel (petroleum ether/EtOAc, 85:15) to give the give the title compound **4d** (40 mg, 53%).

^{1}H NMR (400 MHz, $CDCl_3$) δ 6.83−6.79 (1H, m), 6.75−6.69 (1H, m)),6.65 (1H, d, J = 8.5 Hz), 5.83 (1H, ddt, J = 16.8, 10.2, 6.7 Hz), 5.02 (1H, dd, J = 17.2, 1.5 Hz), 4.95 (1H, d, J = 10.2

Hz), 3.60−3.53 (4H, m), 3.05 (1H, d, *J* = 15.6 Hz), 3.00−2.94 (4H, m), 2.93 (1H, d, *J* = 15.4 Hz), 2.21−2.10 (2H, m), 1.86−1.76 (2H, m), 1.49 (9H, s), 1.42 (3H, s); ^{13}C NMR (100 MHz, $CDCl_3$) δ 154.7, 154.1, 145.5, 138.3, 127.6, 117.8, 115.9, 114.5, 109.2, 88.4, 79.8, 51.6 (x 2), 44.0 (x 2) 41.6, 40.3, 29.6, 28.39, 28.36, 26.4; IR (thin film) 3074, 2972, 1693, 910; HRMS (ESI) exact mass calculated for $C_{22}H_{32}N_2O_3Na$ $[M+Na]^+$ *m/z* 395.2305, found *m/z* 395.2286.

2-(but-3'-en-1'-yl)-*N*,2-dimethyl-*N*-phenyl-2,3-dihydrobenzofuran-5-amine (4e)

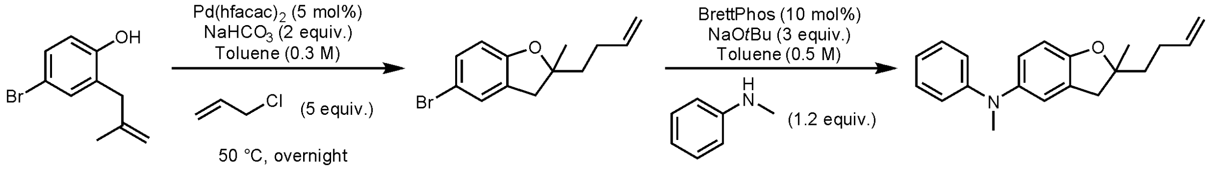

1 2 4e

A 4 mL screw-top glass vial was charged with 4-bromo-2-(2'-methylallyl)phenol (**1**)(45 mg, 0.20 mmol), toluene (0.65 mL), allyl chloride (80 µL, 1.0 mmol), $NaHCO_3$ (34 mg, 0.40 mmol) and Pd(hfacac)$_2$ (5.0 mg, 0.010 mmol) and the vial was sealed under ambient atmosphere. The resulting mixture was heated to 50 °C by immersion of the entire vial into a preheated aluminium block until the substrate had been consumed, as judged by TLC analysis. The reaction mixture was cooled to room temperature and the volatile components were evaporated *in vacuo*. To the vial was added BrettPhos (10 mg, 0.020 mmol), NaO*t*Bu(58 mg, 0.60 mmol) and toluene (0.2 mL). The mixture was allowed to stir for 5 minutes at room temperature. Next, toluene (0.2 mL) and *N*-methylaniline (25 mg, 0.24 mmol) were added and the vial was sealed under ambient atmosphere. The mixture was then heated at 50 °C for 3 h. The reaction mixture was cooled to room temperature, quenched with water and the organic layers were extracted with Et_2O (3 x 20 mL). The combined organic extracts were then washed with brine, dried over $MgSO_4$ and concentrated *in vacuo.* The crude mixture was then purified by flash chromatography on silica gel (petroleum ether/EtOAc; 95:5) to give the title compound **4e** (47 mg, 80%).

^{1}H NMR (500 MHz, $CDCl_3$) δ 7.24−7.18 (2H, m), 7.00−6.97 (1H, m), 6.96−6.91 (1H, m), 6.81−6.76 (3H, m), 6.73 (1H, d, *J* = 8.3 Hz), 5.87 (1H, ddt, *J* = 17.0, 10.3, 6.4 Hz), 5.06 (1H, ddd, *J* = 17.1, 3.4, 1.7 Hz), 4.98 (1H, ddd, *J* = 10.2, 3.0, 1.7 Hz), 3.26 (3H, s), 3.09 (1H, d, *J* = 15.7 Hz), 2.94 (1H, d, *J* = 15.7 Hz), 2.24−2.16 (2H, m), 1.89−1.82 (2H, m), 1.48 (3H, s); ^{13}C NMR (125 MHz, $CDCl_3$) δ 156.2, 149.8, 141.6, 138.3, 128.8 (x 2), 128.1, 125.7, 123.0, 117.9, 115.0 (x 2), 114.6, 109.8, 88.8, 41.3, 40.8, 40.3, 28.4, 26.4; IR (thin film) 3068, 914, 729; HRMS (ESI) exact mass calculated for $C_{20}H_{23}NOH$ $[M+H]^+$ *m/z* 294.1852, found *m/z* 294.1827.

Methyl-2-allyl-benzoate (SI-4)

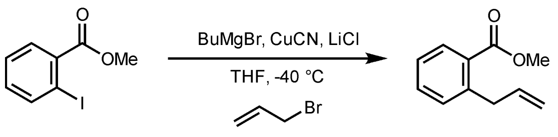

SI-3 SI-4

Following a modification of a reported procedure,[3] to a suspension of magnesium turnings (2.2 g, 92 mmol) and a crystal of iodine in THF (80 mL) was added dropwise bromobutane (9.7 mL, 76 mmol). The mixture was stirred for 15 minutes then cooled to –40 °C before dropwise addition of methyl-2-iodobenzoate (**SI-3**) (5.80 mL, 38.0 mmol). The mixture was stirred at –40 °C for 1.5 h. A freshly prepared solution of LiCl (3.2 g, 76 mmol) and CuCN (3.20 g, 38.0 mmol) in THF (200 mL) was added and the mixture was stirred for a further 15 minutes, followed by the addition of allyl bromide (13.0 mL, 153 mmol). The mixture was stirred at –40 °C for a further 10 minutes, then warmed to room temperature. The mixture was diluted with EtOAc (200 mL) and filtered over Celite®. The filtrate was washed with 25% aq. NH_4OH (200 mL). The aqueous layer was further extracted with EtOAc (2 x 200 mL), and the combined organic extracts washed with brine (200 mL), dried (Na_2SO_4), filtered and concentrated *in vacuo*. Purification by flash chromatography (petroleum ether/EtOAc; 9:1), afforded methyl-2-allyl-benzoate (**SI-4**) as a colourless oil (6.39 g, 95%). Data corresponded to literature values.[3]

2-allylbenzoic acid (SI-5)

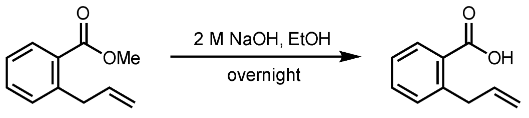

SI-4 SI-5

Methyl 2-allylbenzoate (**SI-4**) (528 mg, 3.00 mmol) was dissolved in EtOH (10 mL), and 2 M aq. NaOH (10 mL) added. The mixture was stirred for at room temperature overnight then EtOH was removed *in vacuo.* The residue was extracted with Et_2O (2 x 10 mL), acidified to pH 1 with 2 M aq. HCl and extracted with EtOAc (3 x 10 mL). The combined organic layers were dried ($MgSO_4$), filtered and concentrated *in vacuo* to afford 2-allylbenzoic acid(**SI-5**) as a colourless solid (400 mg, 82%), which was used directly without further purification. Data corresponded to literature values.[3]

2-allyl-*N*-tosylbenzamide (5a)

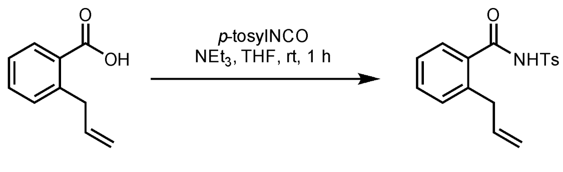

SI-5 5a

To a solution of 2-allyl benzoic acid (**SI-5**) (2.35 g, 14.5 mmol) in THF (43 mL) was added *p*-tosyl isocyanate (2.20 g, 14.5 mmol). The resulting solution was stirred for 10 minutes then triethylamine (2.02 mL, 14.5 mmol) added dropwise. Gas evolution was observed on addition. After 1 h, the mixture was diluted with EtOAc (50 mL) and washed with 2 M aq. HCl (50 mL) and brine (50 mL). The organic extracts were dried ($MgSO_4$), filtered and concentrated *in vacuo*. The crude product was purified by column chromatography (CH_2Cl_2) to afford the title compound (**5a**) as a colourless solid (3.68 g, 81%). Data corresponded to literature values.[3]

1-allyl-*N*-tosyl-pyrrole-2-carboxamide (5b)

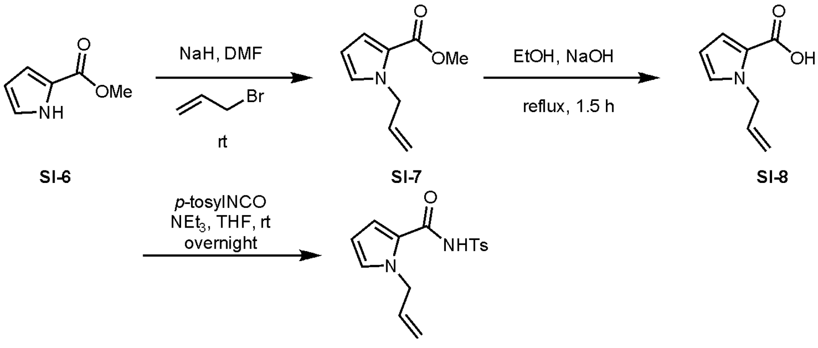

5b

Following a modification of a reported procedure,[3] to a cooled (0 °C) suspension of NaH (60% dispersion in mineral oil, 480 mg, 12 mmol) in DMF (10 mL) was added a solution of methyl-2-pyrrole-carboxylate (**SI-6**) (1.0 g, 8.0 mmol) in DMF (2 mL). The resulting mixture was stirred for 20 min at 0 °C, then allyl bromide (1.2 mL, 14 mmol) added dropwise. The mixture was allowed to warm to room temperature, and stirred for 2 h, then quenched by pouring onto ice (30 g). The mixture was extracted with Et_2O (3 x 15 mL) and the combined organic extracts were washed with water (4 x 15 mL), brine (1 x 15 mL), dried ($MgSO_4$), filtered and concentrated *in vacuo* to afford methyl-1-allyl-pyrrole-2-carboxylate (**SI-7**) as a colourless oil (1.3 g, 98%) which was used directly without further purification.

To a solution of methyl-1-allyl-pyrrole-2-carboxylate (**SI-7**) (1.30 g, 7.87 mmol) in EtOH (20 mL) was added 1 M aq. NaOH (20 mL). The mixture was refluxed for 1.5 h, then EtOH was removed *in vacuo*. The aqueous phase was washed with EtOAc (3 x 25 mL) then acidified to pH 2 with 4 M aq. HCl and extracted with EtOAc (3 x 25 mL). The combined organic extracts

were dried ($MgSO_4$), filtered and concentrated *in vacuo*, to afford 1-allyl-pyrrole-2-carboxylic acid (**SI-8**) as a colourless solid (1.0 g, 89%) which was used directly without further purification.

To a flask charged with 1-allyl-pyrrole-2-carboxylic acid (**SI-8**) (0.50 g, 3.3 mmol) in THF (10 mL) was added *p*-tosyl isocyanate (0.77 g, 6.6 mmol). The resulting solution was stirred for 10 minutes then triethylamine (0.50 mL, 3.6 mmol) added dropwise. Gas evolution was observed on addition. After 1 h, the mixture was diluted with EtOAc (50 mL) and washed with 2 M aq. HCl (50 mL) and brine (50 mL). The organic extracts were dried ($MgSO_4$), filtered and concentrated *in vacuo*. The crude product was purified by column chromatography (CH_2Cl_2) to afford the title compound (**5b**) as a colourless solid (0.71 g, 61%). Data corresponded to literature values.[3]

3-(3'-oxobutyl)-2-tosyl-3,4-dihydroisoquinolinone (6a)

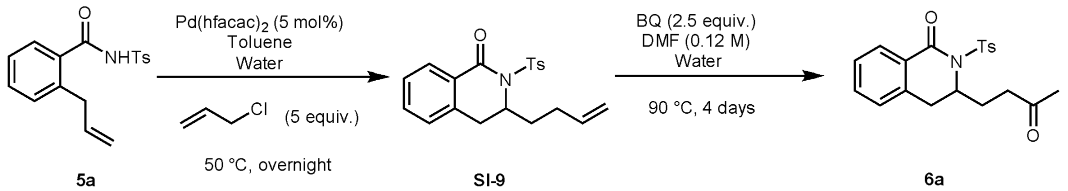

A 4 mL screw-top glass vial was charged with 2-allyl-*N*-tosyl-benzamide (**5a**) (50 mg, 0.16 mmol), toluene (0.64 mL), H_2O (0.64 mL), allyl chloride (0.070 mL, 0.79 mmol) and Pd(hfacac)$_2$ (4.0 mg, 0.01 mmol). The mixture was heated at 50 °C overnight. The reaction mixture was cooled to room temperature and the volatile components were evaporated *in vacuo*. Following a modification of a literature procedure,[4] to the mixture was added *p*-benzoquinone (43 mg, 0.4 mmol), DMF (0.53 mL) and water (50 µL). The vial was then sealed and heated at 90 °C for four days. The mixture was allowed to cool to room temperature before being poured into water and the organic layers were extracted with EtOAc (3 x 20 mL), dried over $MgSO_4$, and concentrated *in vacuo*. The crude mixture was then purified by flash chromatography on silica gel (toluene/Et_2O; 9:1) to give the title compound (**6a**) (32 mg, 54%).

^{1}H NMR (400 MHz, $CDCl_3$) δ 7.99−7.92 (3H, m), 7.48 (1H, td, *J* = 7.4, 1.3 Hz), 7.34−7.28 (3H, m), 7.20 (1H, d, *J* = 7.5 Hz), 5.00−4.93 (1H, m), 3.38 (1H, dd, *J* = 16.2, 5.5 Hz), 2.94 (1H, dd, *J*=16.2, 1.9 Hz), 2.81 (1H, dt, *J* = 18.3, 7.7 Hz), 2.60 (1H, dt, *J* = 18.3, 6.5 Hz), 2.41 (3H, s), 2.18 (3H, s), 1.89−1.82 (2H, m); ^{13}C NMR (100 MHz, $CDCl_3$) δ 207.6, 162.6, 144.8, 136.9, 136.3, 133.7, 129.4 (x 2), 128.9, 128.8 (x 2), 128.2, 127.9, 127.5, 55.0, 39.8, 34.1, 30.0, 27.7, 21.6; IR (thin film) 2958, 1710, 1685, 736; HRMS (ESI) exact mass calculated for $C_{20}H_{21}NO_4SNa$ $[M+Na]^+$ *m/z* 394.1083, found *m/z* 394.1072.

Oxidised pyrrole tosylamide (6b)

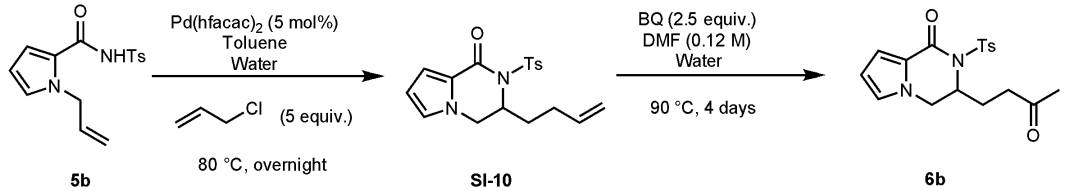

5b **SI-10** **6b**

A 4 mL glass screw-top vial was charged with 1-allyl-*N*-tosyl-pyrrole-2-carboxamide (**5b**) (48 mg, 0.16 mmol), toluene (0.64 mL), H_2O (0.64 mL), allyl chloride (0.07 mL, 0.79 mmol) and $Pd(hfacac)_2$ (4.0 mg, 0.01 mmol). The mixture was heated at 80 °C for 19 h. The reaction mixture was cooled to room temperature and the volatile components were evaporated *in vacuo*. Following a modification of a literature procedure,[4] to the mixture was added *p*-benzoquinone (43 mg, 0.40 mmol), DMF (0.53 mL) and water (50 μL). The vial was then sealed and heated at 90 °C for four days. The mixture was allowed to cool to room temperature before being poured into water and the organic layers were extracted with EtOAc (3 x 20 mL), dried over $MgSO_4$, and concentrated *in vacuo*. The crude mixture was then purified by flash chromatography on silica gel (toluene/EtOAc; 8:2) to give the title compound (**6b**) (28 mg, 48%).

^{1}H NMR (400 MHz, $CDCl_3$) δ 7.95 (2H, d, *J* = 8.4 Hz), 7.31 (2H, d, *J* = 8.4 Hz), 6.97 (1H, dd, *J* = 4.0, 1.5 Hz), 6.78−6.75 (1H, m), 6.23 (1H, dd, *J* = 3.9, 2.4 Hz), 5.01−4.93 (1H, m), 4.29 (1H, dd, *J* = 13.2, 4.1 Hz), 4.13 (1H, dd, *J* = 13.2, 1.5 Hz), 2.86 (1H, ddd, *J* = 18.7, 8.3, 6.5 Hz), 2.59 (1H, dt, *J* = 18.7, 6.5 Hz), 2.42 (3H, s), 2.19 (3H, s), 2.05−1.93 (1H, m), 1.91−1.73 (1H, m); ^{13}C NMR (100 MHz, $CDCl_3$) δ 207.5, 155.8, 144.9, 136.1, 129.4 (x 2), 128.9 (x 2), 125.3, 122.3, 116.9, 111.1, 55.1, 49.0, 39.2, 30.0, 26.9, 21.6; IR (thin film) 3250, 1723, 1674, 731; HRMS (ESI) exact mass calculated for $C_{18}H_{20}N_2O_4SNa$ $[M+Na]^+$ *m/z* 383.1036, found *m/z* 383.1032.

4-(1'-oxo-2'-tosyl-1',2',3',4'-tetrahydroisoquinolin-3'-yl)butanal (7)

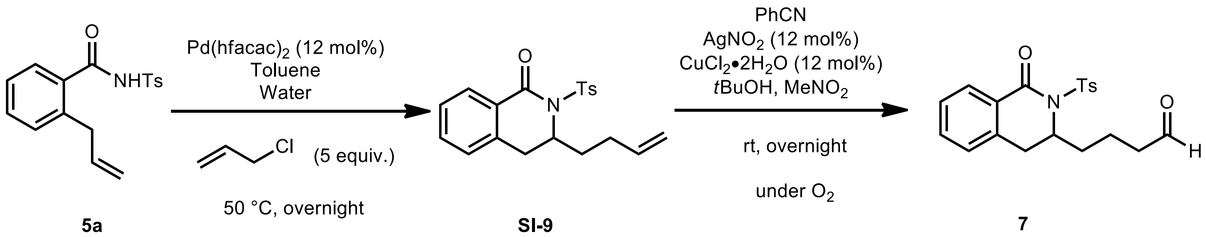

5a **SI-9** **7**

A 4 mL screw-top glass vial was charged with 2-allyl-*N*-tosyl-benzamide (**5a**) (63 mg, 0.20 mmol), toluene (0.65 mL), H_2O (0.65 mL), allyl chloride (0.080 mL, 1.0 mmol) and

$Pd(hfacac)_2$ (13 mg, 0.024 mmol). The mixture was heated at 50 °C for 16 h. The reaction mixture was cooled to room temperature and the volatile components were evaporated *in vacuo*. Following a modification of a literature procedure,[5] to the vial was added benzonitrile (50 µL, 0.48 mmol), $CuCl_2•2H_2O$ (4.0 mg, 0.024 mmol) and $AgNO_2$ (4.0 mg, 0.024 mmol). The vial was sparged for 45 seconds with oxygen (1 atm balloon) then subsequently *t*BuOH (3 mL), $MeNO_2$ (0.2 mL) were added *via* syringe. The solution was saturated with oxygen by an additional 45 seconds of sparging. The reaction was then allowed to stir at room temperature overnight. Next, the reaction was quenched by addition to water and extracted three times with Et_2O (3 x 20 mL). The combined organic layers were subsequently dried over $MgSO_4$. Immediately prior to NMR analysis trimethoxybenzene (34 mg, 0.2 mmol) was added as an internal standard. The resulting solution was subsequently subjected to ^1H NMR analysis to determine yield (33%) of aldehyde (**7**) and (19%) ketone (**6a**). The title compound (**7**) was obtained using flash chromatography on silica gel (toluene/EtOAc; 9:1).

Data for aldehyde (**7**):

^1H NMR (400 MHz, $CDCl_3$) δ 9.74 (1H, t, *J* = 1.3 Hz), 8.02−7.94 (3H, m), 7.49 (1H, td, *J* = 7.5, 1.3 Hz), 7.35−7.30 (3H, m), 7.21 (1H, d, *J* = 7.5 Hz), 5.00−4.89 (1H, m), 3.37 (1H, dd, *J* = 16.2, 5.4 Hz), 2.98 (1H, dd, *J* = 16.2, 1.8 Hz), 2.50−2.39 (5H, m),1.81−1.68 (3H, m), 1.64−1.54 (1H, m); ^{13}C NMR (125 MHz, $CDCl_3$) δ 201.5, 162.6, 144.8, 136.6, 136.5, 133.7, 129.3 (x 2), 128.9 (x 3), 128.2, 128.0, 127.5, 55.3, 43.2, 33.1, 32.5, 21.6, 18.8; IR (thin film) 1722, 1683, 906, 725; HRMS (ESI) exact mass calculated for $C_{20}H_{21}NO_4SNa$ $[M+Na]^+$ *m/z* 394.1083, found *m/z* 394.1087.

Data for ketone (**6a**):

^1H NMR (400 MHz, $CDCl_3$) δ 7.99−7.92 (3H, m), 7.48 (1H, td, *J* = 7.4, 1.3 Hz), 7.34−7.28 (3H, m), 7.20 (1H, d, *J* = 7.5 Hz), 5.00−4.93 (1H, m), 3.38 (1H, dd, *J* = 16.2, 5.5 Hz), 2.94 (1H, dd, *J*=16.2, 1.9 Hz), 2.81 (1H, dt, *J* = 18.3, 7.7 Hz), 2.60 (1H, dt, *J* = 18.3, 6.5 Hz), 2.41 (3H, s), 2.18 (3H, s), 1.89−1.82 (2H, m); ^{13}C NMR (100 MHz, $CDCl_3$) δ 207.6, 162.6, 144.8, 136.9, 136.3, 133.7, 129.4 (x 2), 128.9, 128.8 (x 2), 128.2, 127.9, 127.5, 55.0, 39.8, 34.1, 30.0, 27.7, 21.6; IR (thin film) 2958, 1710, 1685, 736; HRMS (ESI) exact mass calculated for $C_{20}H_{21}NO_4SNa$ $[M+Na]^+$ *m/z* 394.1083, found *m/z* 394.1072.

References

[1] S. Nicolai, S. Erard, D. F. González, J. Waser, *Org. Lett.* **2010**, *12*, 384–387.

[2] X.-L. Fu, L.-L. Wu, H.-Y. Fu, H. Chen, R.-X. Li, *Eur. J. Org. Chem.* **2009**, *2009*, 2051–2054.

[3] S. Nicolai, C. Piemontesi, J. Waser, *Angew. Chem. Int. Ed.* **2011**, *50*, 4680–4683.

[4] H. Someya, H. Yorimitsu, K. Oshima, *Tetrahedron Lett.* **2009**, *50*, 3270–3272.

[5] Z. K. Wickens, B. Morandi, R. H. Grubbs, *Angew. Chem. Int. Ed.* **2013**, *52*, 11257–11260.

user Alina Tirla
AT046 P
PROTON.GLA CDCl3 /u alina 20

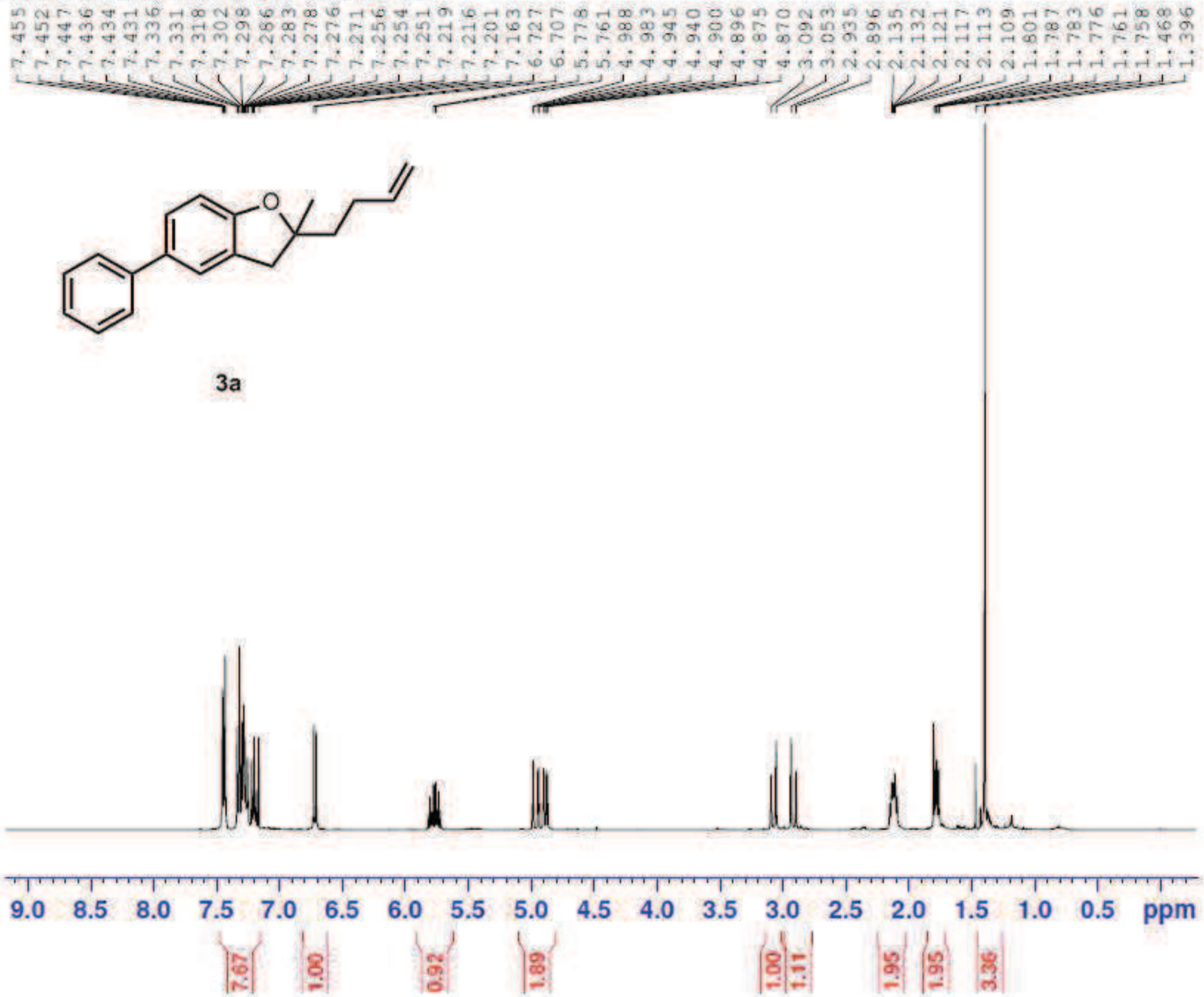

Current Data Parameters
NAME AT046
EXPNO 10
PROCNO 1

F2 - Acquisition Parameters
Date_ 20150130
Time 16.01
INSTRUM spect
PROBHD 5 mm PABBO BB-
PULPROG zg30
TD 74012
SOLVENT CDCl3
NS 16
DS 2
SWH 8223.685 Hz
FIDRES 0.111113 Hz
AQ 4.4999294 sec
RG 80.6
DW 60.800 usec
DE 16.87 usec
TE 294.5 K
D1 0.50000000 sec
TD0 1

======== CHANNEL f1 ========
SFO1 400.1924713 MHz
NUC1 1H
P1 10.00 usec
PLW1 23.03800011 W

F2 - Processing parameters
SI 131072
SF 400.1900488 MHz
WDW EM
SSB 0
LB 0.30 Hz
GB 0
PC 1.00

user Alina Tirla
AT006
C13CPD1024.GLA CDCl3 /u alina 45

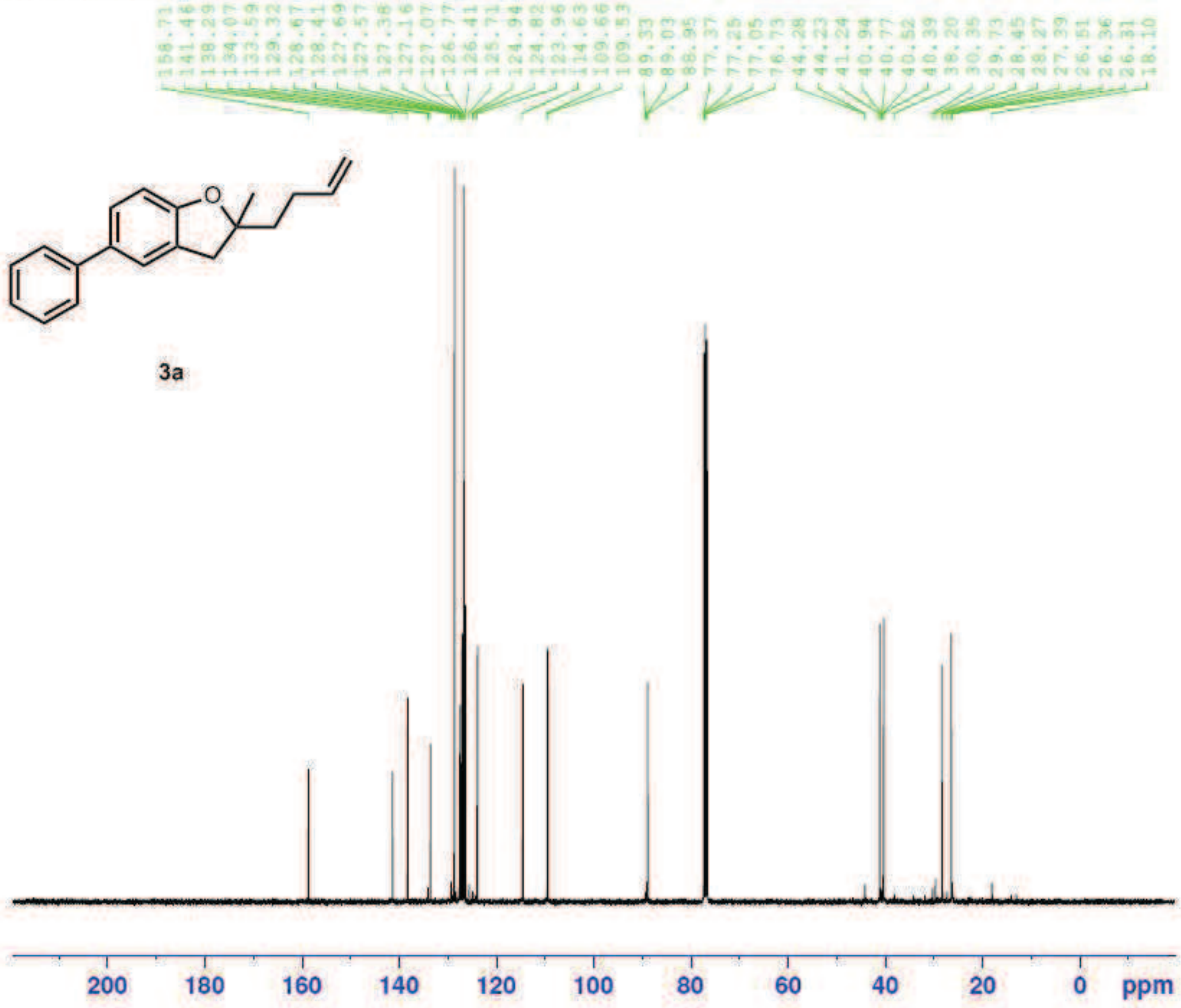

Current Data Parameters
NAME AT006
EXPNO 13
PROCNO 1

F2 - Acquisition Parameters
Date_ 20141010
Time 4.32
INSTRUM spect
PROBHD 5 mm PABBO BB-
PULPROG zgpg30
TD 65536
SOLVENT CDCl3
NS 1024
DS 4
SWH 24038.461 Hz
FIDRES 0.366798 Hz
AQ 1.3631488 sec
RG 2050
DW 20.800 usec
DE 9.78 usec
TE 299.6 K
D1 2.00000000 sec
D11 0.03000000 sec
TD0 1

======== CHANNEL f1 ========
SFO1 100.6379183 MHz
NUC1 13C
P1 9.00 usec
PLW1 51.32600021 W

======== CHANNEL f2 ========
SFO2 400.1916008 MHz
NUC2 1H
CPDPRG[2 waltz16
PCPD2 90.00 usec
PLW2 26.45100021 W
PLW12 0.26451001 W
PLW13 0.21425000 W

F2 - Processing parameters
SI 32768
SF 100.6278560 MHz
WDW EM
SSB 0
LB 1.00 Hz
GB 0
PC 1.40

user Alina Tirla
AT045 P
PROTON.GLA CDCl3 /u alina 44

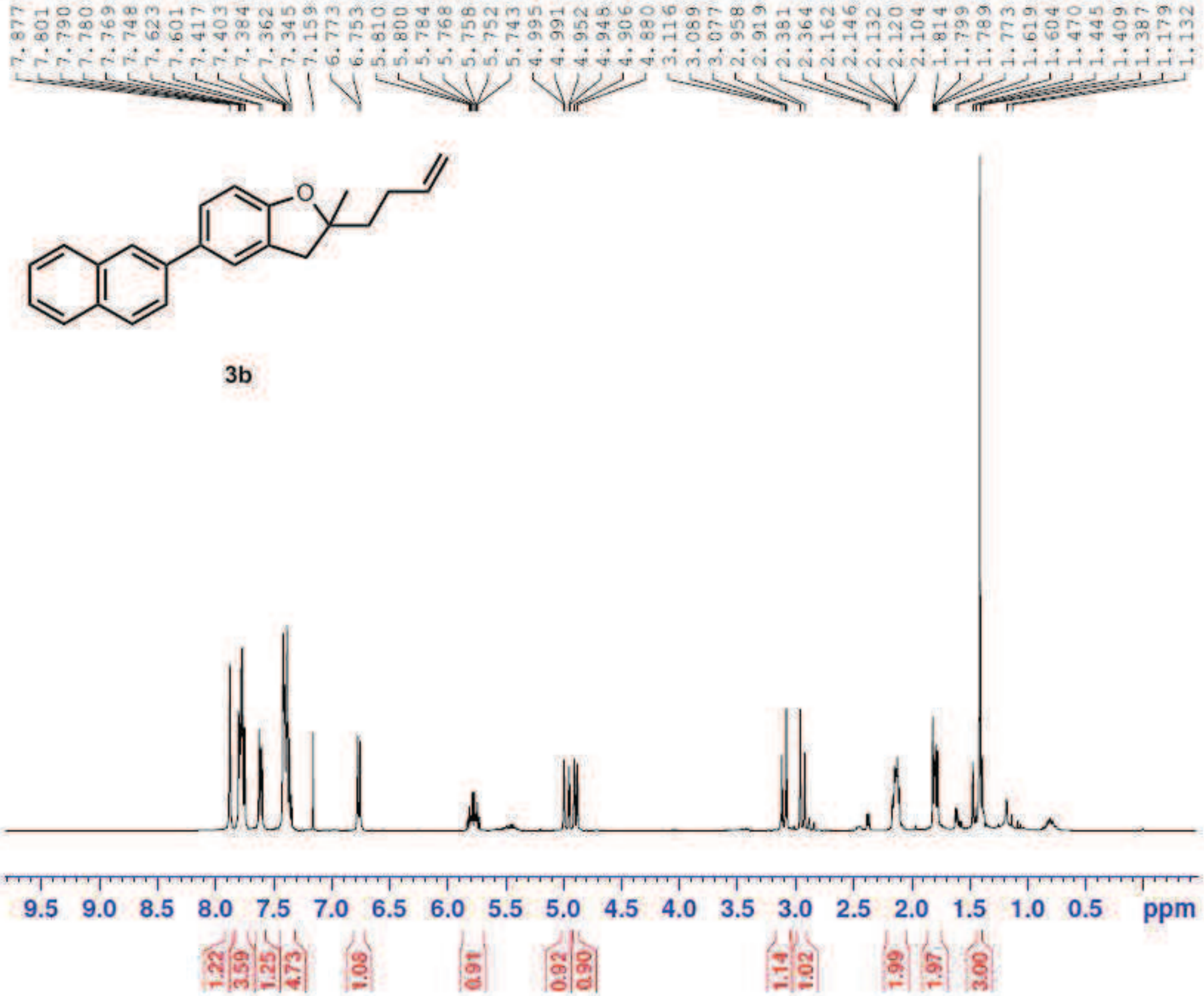

Current Data Parameters
NAME AT045
EXPNO 10
PROCNO 1

F2 - Acquisition Parameters
Date_ 20150130
Time 12.30
INSTRUM spect
PROBHD 5 mm PABBO BB-
PULPROG zg30
TD 74012
SOLVENT CDCl3
NS 16
DS 2
SWH 8223.685 Hz
FIDRES 0.111113 Hz
AQ 4.4999294 sec
RG 80.6
DW 60.800 usec
DE 16.87 usec
TE 294.3 K
D1 0.50000000 sec
TD0 1

======== CHANNEL f1 ========
SFO1 400.1924713 MHz
NUC1 1H
P1 10.00 usec
PLW1 23.03800011 W

F2 - Processing parameters
SI 131072
SF 400.1900507 MHz
WDW EM
SSB 0
LB 0.30 Hz
GB 0
PC 1.00

user Alina Tirla
C13CPD1024.GLA CDCl3 /u alina 42

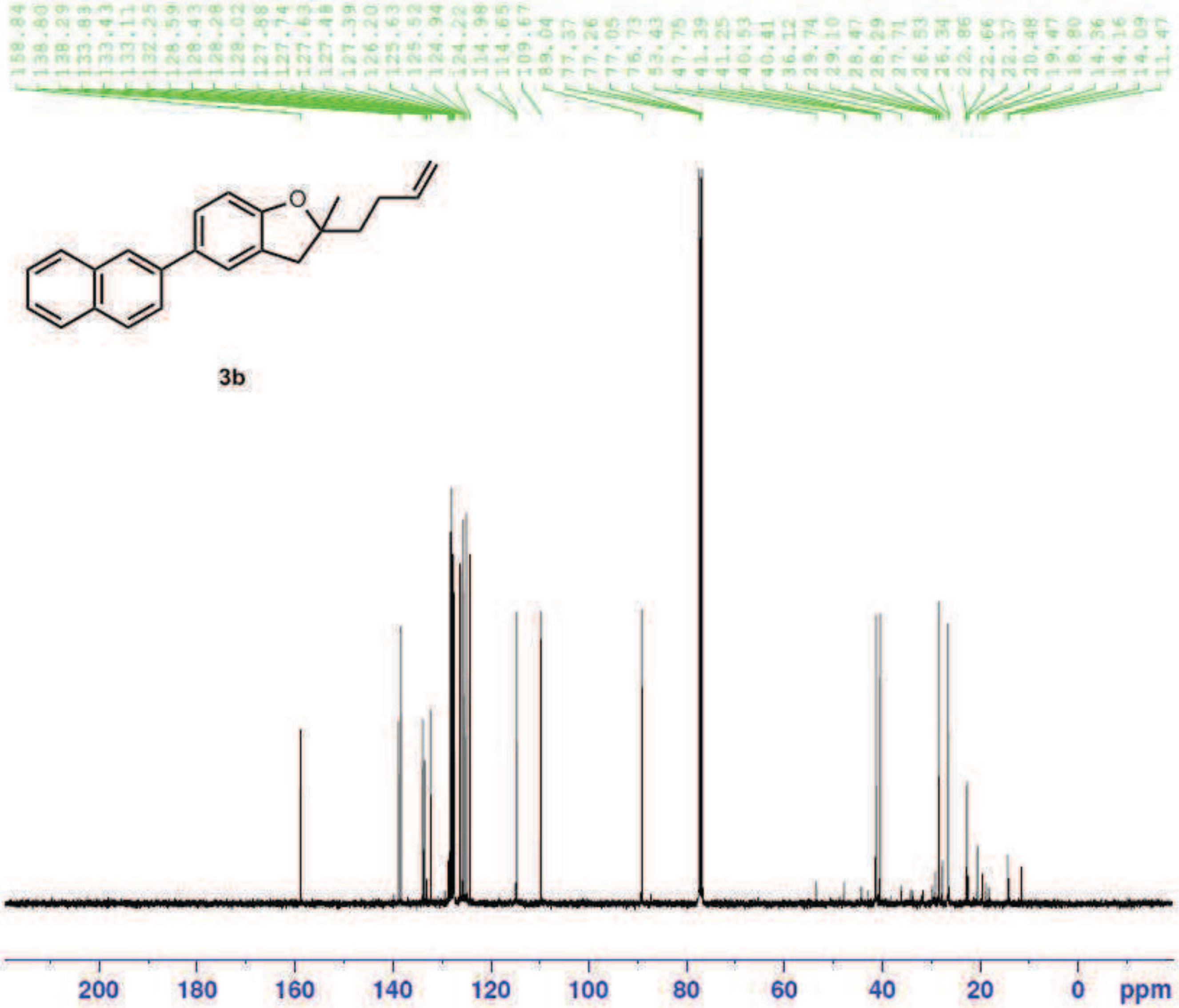


```
Current Data Parameters
NAME                AT024
EXPNO                  40
PROCNO                  1

F2 - Acquisition Parameters
Date_            20141113
Time                23.55
INSTRUM             spect
PROBHD   5 mm PABBO BB-
PULPROG           zgpg30
TD                  65536
SOLVENT             CDCl3
NS                   1024
DS                      4
SWH             24038.461 Hz
FIDRES           0.366798 Hz
AQ              1.3631488 sec
RG                   2050
DW                 20.800 usec
DE                   9.78 usec
TE                  298.3 K
D1             2.00000000 sec
D11            0.03000000 sec
TD0                     1

======== CHANNEL f1 ========
SFO1          100.6379183 MHz
NUC1                  13C
P1                   9.00 usec
PLW1          51.32600021 W

======== CHANNEL f2 ========
SFO2          400.1916008 MHz
NUC2                   1H
CPDPRG[2          waltz16
PCPD2               90.00 usec
PLW2          26.45100021 W
PLW12          0.26451001 W
PLW13          0.21425000 W

F2 - Processing parameters
SI                  32768
SF            100.6278560 MHz
WDW                    EM
SSB       0
LB                   1.00 Hz
GB        0
PC                   1.40
```

user Craig Smith
PROTON.GLA CDCl3 /u craigsm 31

3c

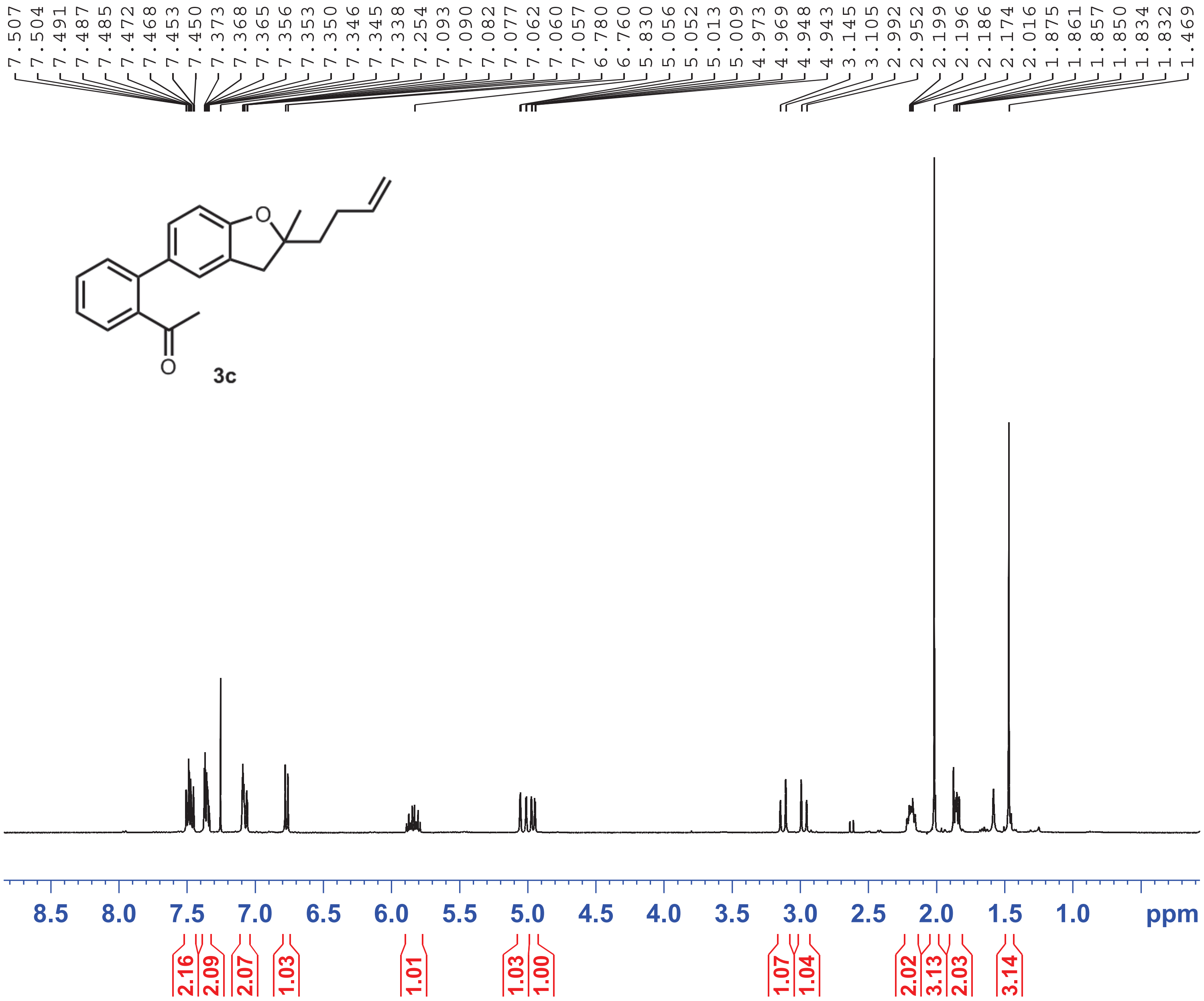

Current Data Parameters
NAME CDS-IX-66A
EXPNO 10
PROCNO 1

F2 - Acquisition Parameters
Date_ 20151105
Time 10.56
INSTRUM spect
PROBHD 5 mm PABBO BB-
PULPROG zg30
TD 74012
SOLVENT CDCl3
NS 16
DS 2
SWH 8223.685 Hz
FIDRES 0.111113 Hz
AQ 4.4999294 sec
RG 228
DW 60.800 usec
DE 16.87 usec
TE 297.2 K
D1 0.50000000 sec
TD0 1

======== CHANNEL f1 ========
SFO1 400.1924713 MHz
NUC1 1H
P1 10.00 usec
PLW1 23.03800011 W

F2 - Processing parameters
SI 131072
SF 400.1900130 MHz
WDW EM
SSB 0
LB 0.30 Hz
GB 0
PC 1.00

user Craig Smith
C13CPD1024.GLA CDCl3 /u craigsm 31

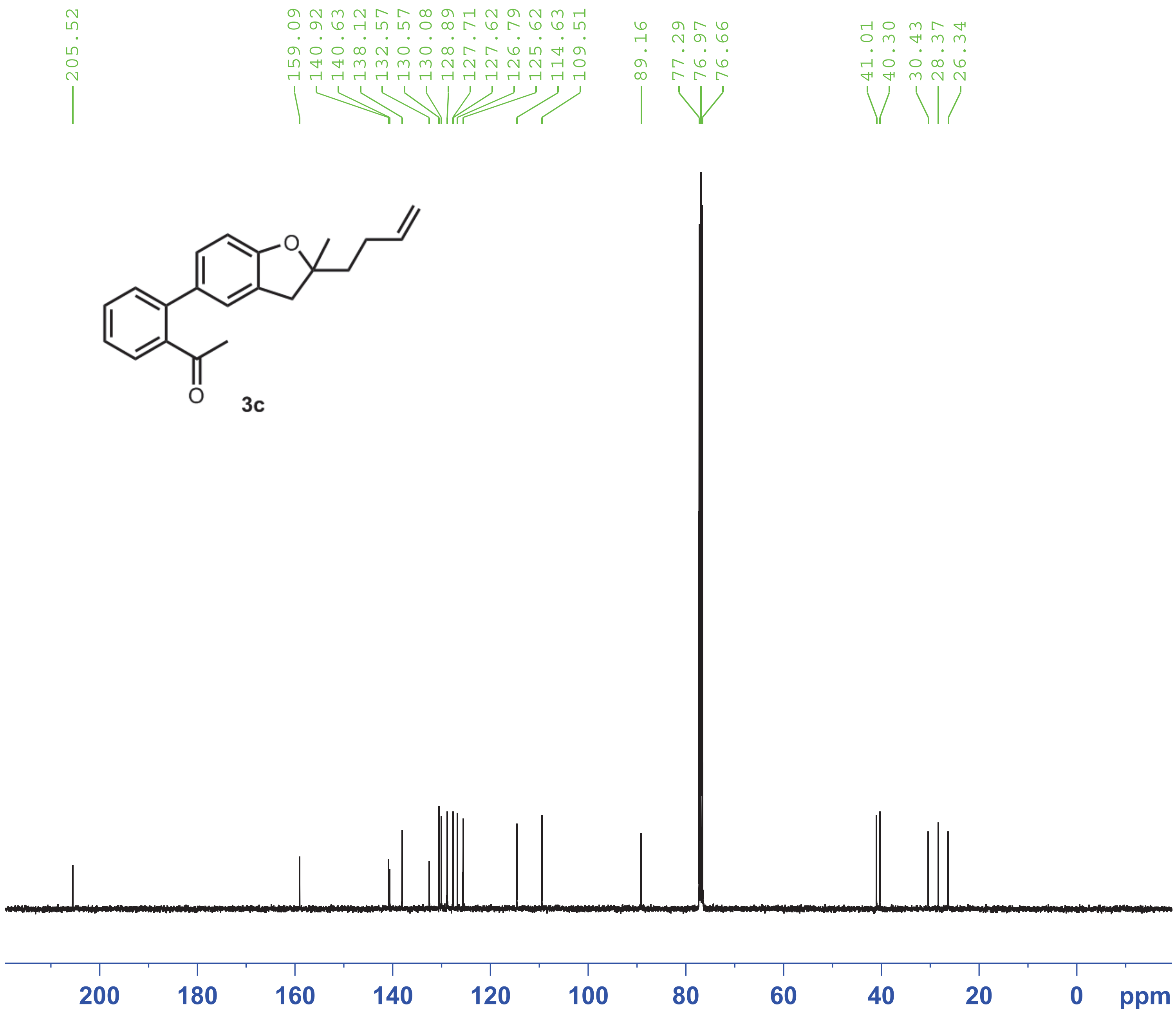

Current Data Parameters
NAME CDS-IX-66A
EXPNO 25
PROCNO 1

F2 - Acquisition Parameters
Date_ 20151106
Time 23.55
INSTRUM spect
PROBHD 5 mm PABBO BB-
PULPROG zgpg30
TD 65536
SOLVENT CDCl3
NS 1024
DS 4
SWH 24038.461 Hz
FIDRES 0.366798 Hz
AQ 1.3631488 sec
RG 2050
DW 20.800 usec
DE 9.78 usec
TE 298.0 K
D1 2.00000000 sec
D11 0.03000000 sec
TD0 1

======== CHANNEL f1 ========
SFO1 100.6379183 MHz
NUC1 13C
P1 9.00 usec
PLW1 51.32600021 W

======== CHANNEL f2 ========
SFO2 400.1916008 MHz
NUC2 1H
CPDPRG[2 waltz16
PCPD2 90.00 usec
PLW2 26.45100021 W
PLW12 0.26451001 W
PLW13 0.21425000 W

F2 - Processing parameters
SI 32768
SF 100.6278606 MHz
WDW EM
SSB 0
LB 1.00 Hz
GB 0
PC 1.40

user Alina Tirla
AT060
PROTON.GLA CDCl3 /u alina 59

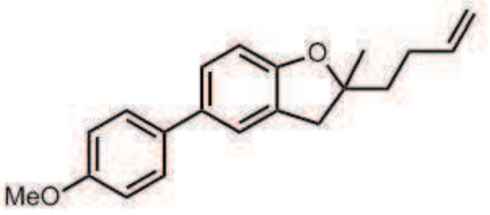

3d

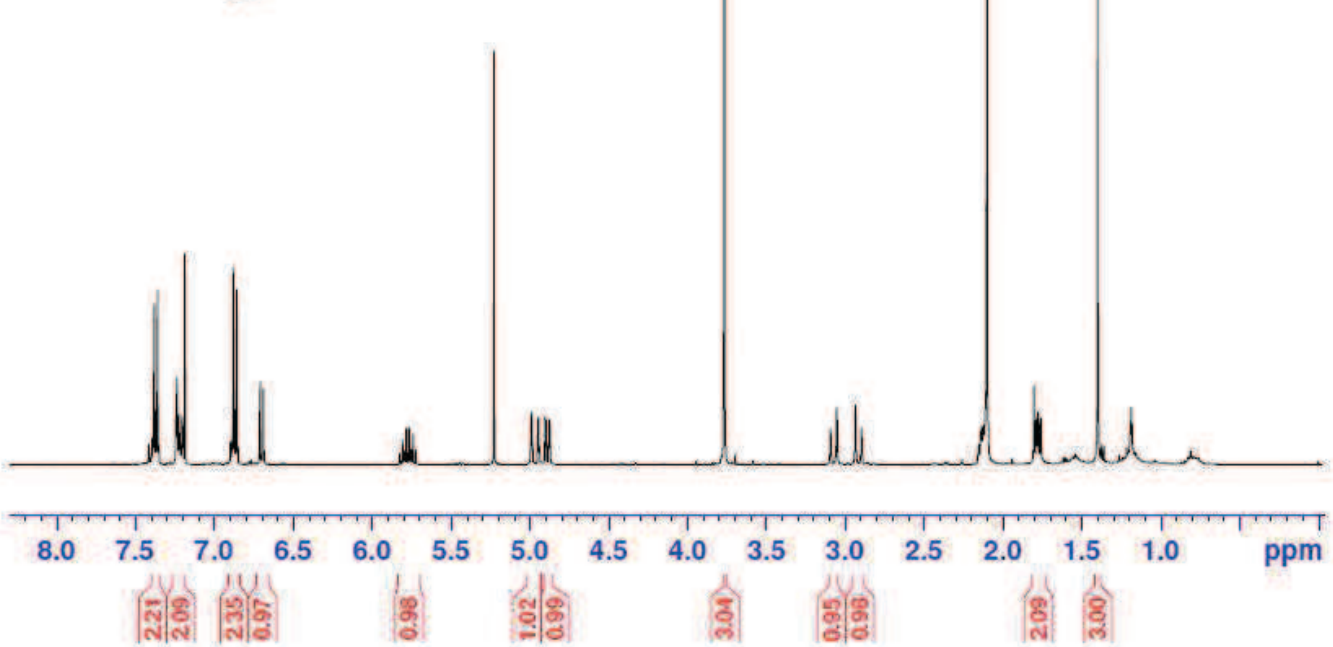

Current Data Parameters
NAME AT060
EXPNO 40
PROCNO 1

F2 - Acquisition Parameters
Date_ 20150222
Time 10.53
INSTRUM spect
PROBHD 5 mm PABBO BB-
PULPROG zg30
TD 74012
SOLVENT CDCl3
NS 16
DS 2
SWH 8223.685 Hz
FIDRES 0.111113 Hz
AQ 4.4999294 sec
RG 181
DW 60.800 usec
DE 16.87 usec
TE 294.5 K
D1 0.50000000 sec
TD0 1

======== CHANNEL f1 ========
SFO1 400.1924713 MHz
NUC1 1H
P1 10.00 usec
PLW1 23.03800011 W

F2 - Processing parameters
SI 131072
SF 400.1900390 MHz
WDW EM
SSB 0
LB 0.30 Hz
GB 0
PC 1.00

user Alina Tirla
AT060
C13CPD1024.GLA CDCl3 /u alina 59

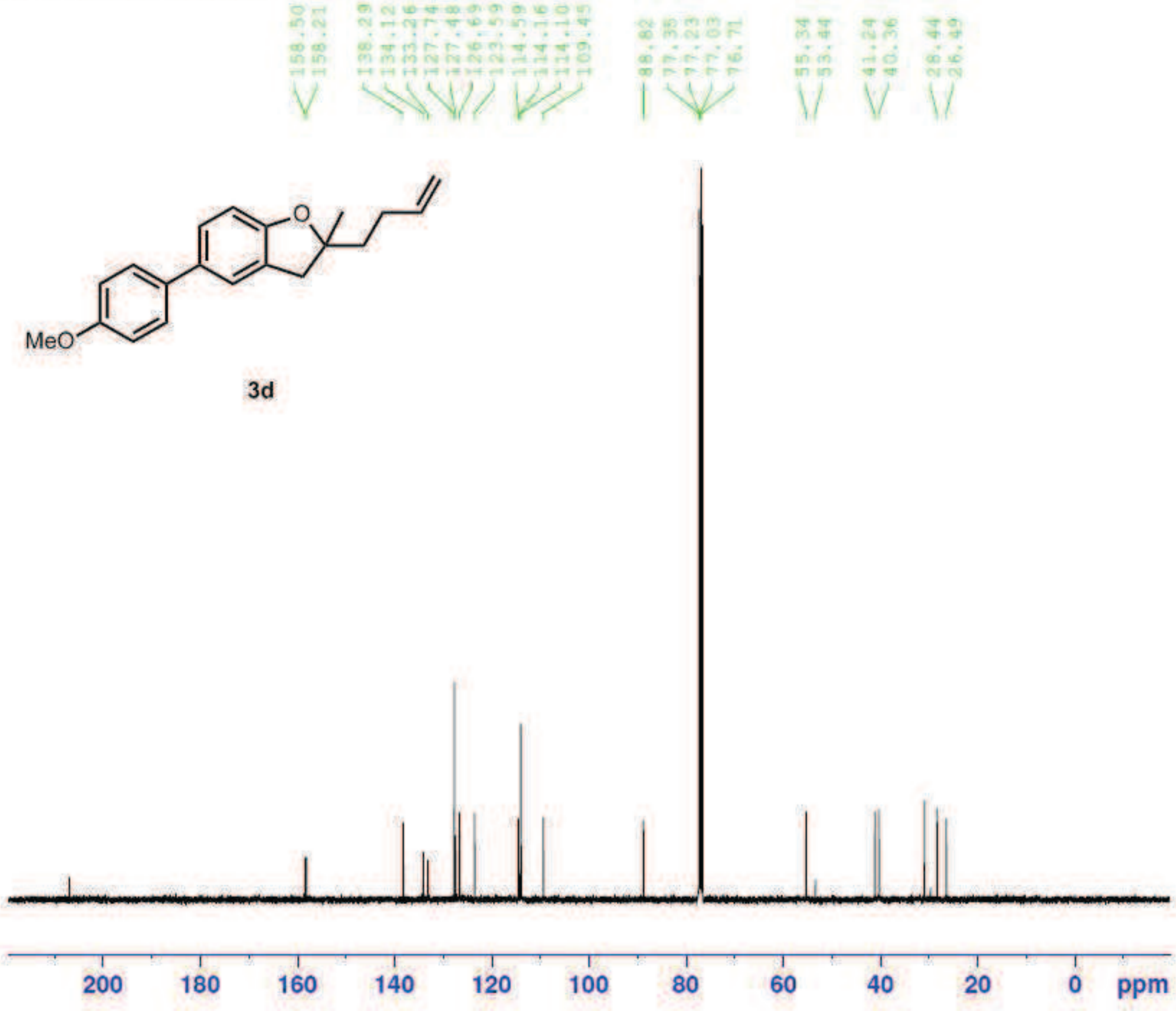

3d

Current Data Parameters
NAME AT060
EXPNO 44
PROCNO 1

F2 - Acquisition Parameters
Date_ 20150222
Time 12.39
INSTRUM spect
PROBHD 5 mm PABBO BB-
PULPROG zgpg30
TD 65536
SOLVENT CDCl3
NS 1024
DS 4
SWH 24038.461 Hz
FIDRES 0.366798 Hz
AQ 1.3631488 sec
RG 2050
DW 20.800 usec
DE 9.78 usec
TE 295.1 K
D1 2.00000000 sec
D11 0.03000000 sec
TD0 1

======== CHANNEL f1 ========
SFO1 100.6379183 MHz
NUC1 13C
P1 9.00 usec
PLW1 51.32600021 W

======== CHANNEL f2 ========
SFO2 400.1916008 MHz
NUC2 1H
CPDPRG[2 waltz16
PCPD2 90.00 usec
PLW2 26.45100021 W
PLW12 0.26451001 W
PLW13 0.21425000 W

F2 - Processing parameters
SI 32768
SF 100.6278560 MHz
WDW EM
SSB 0
LB 1.00 Hz
GB 0
PC 1.40

user Alina Tirla
PROTON.GLA CDCl3 /u alina 21

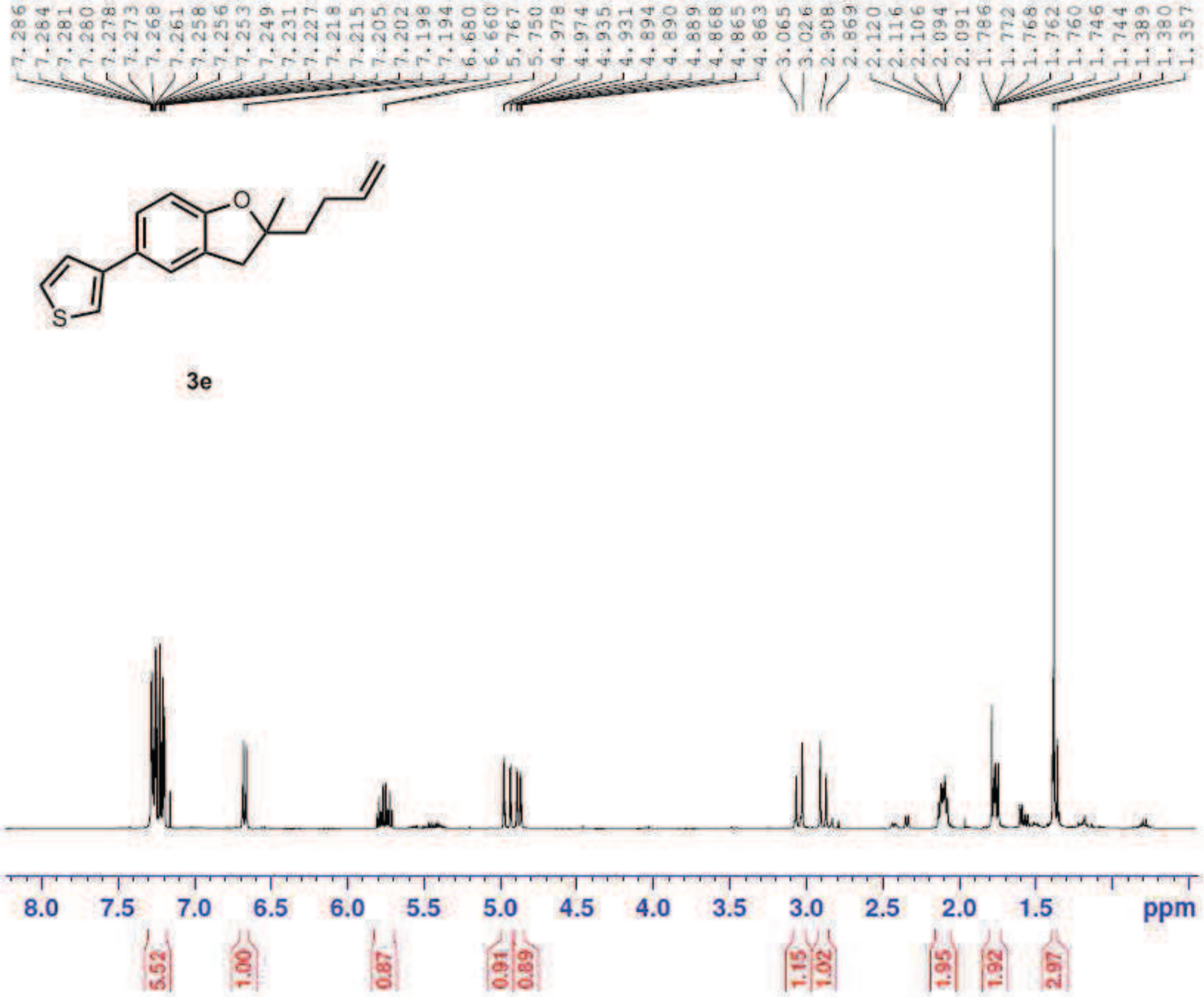

3e

Current Data Parameters
NAME AT059
EXPNO 40
PROCNO 1

F2 - Acquisition Parameters
Date_ 20150221
Time 19.43
INSTRUM spect
PROBHD 5 mm PABBO BB-
PULPROG zg30
TD 74012
SOLVENT CDCl3
NS 16
DS 2
SWH 8223.685 Hz
FIDRES 0.111113 Hz
AQ 4.4999294 sec
RG 80.6
DW 60.800 usec
DE 16.87 usec
TE 294.8 K
D1 0.50000000 sec
TD0 1

======== CHANNEL f1 ========
SFO1 400.1924713 MHz
NUC1 1H
P1 10.00 usec
PLW1 23.03800011 W

F2 - Processing parameters
SI 131072
SF 400.1900498 MHz
WDW EM
SSB 0
LB 0.30 Hz
GB 0
PC 1.00

user Alina Tirla
C13CPD1024.GLA CDCl3 /u alina 21

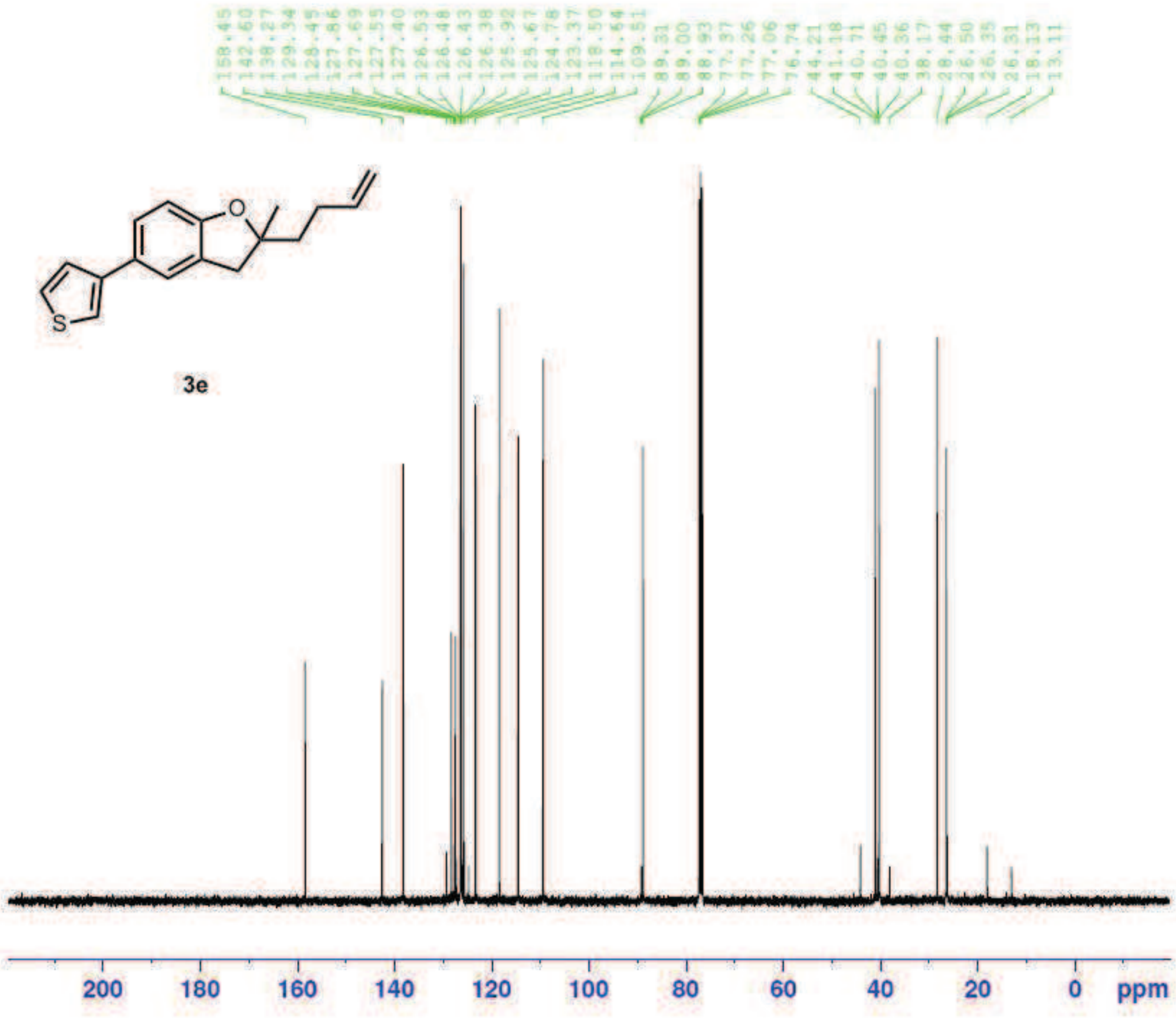

Current Data Parameters
NAME AT059
EXPNO 44
PROCNO 1

F2 - Acquisition Parameters
Date_ 20150221
Time 21.29
INSTRUM spect
PROBHD 5 mm PABBO BB-
PULPROG zgpg30
TD 65536
SOLVENT CDCl3
NS 1024
DS 4
SWH 24038.461 Hz
FIDRES 0.366798 Hz
AQ 1.3631488 sec
RG 2050
DW 20.800 usec
DE 9.78 usec
TE 295.4 K
D1 2.00000000 sec
D11 0.03000000 sec
TD0 1

======== CHANNEL f1 ========
SFO1 100.6379183 MHz
NUC1 13C
P1 9.00 usec
PLW1 51.32600021 W

======== CHANNEL f2 ========
SFO2 400.1916008 MHz
NUC2 1H
CPDPRG[2 waltz16
PCPD2 90.00 usec
PLW2 26.45100021 W
PLW12 0.26451001 W
PLW13 0.21425000 W

F2 - Processing parameters
SI 32768
SF 100.6278560 MHz
WDW EM
SSB 0
LB 1.00 Hz
GB 0
PC 1.40

user Craig Smith
PROTON.GLA CDCl3 /u craigsm 33

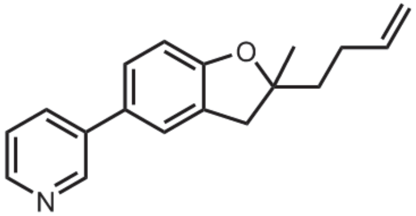

3f

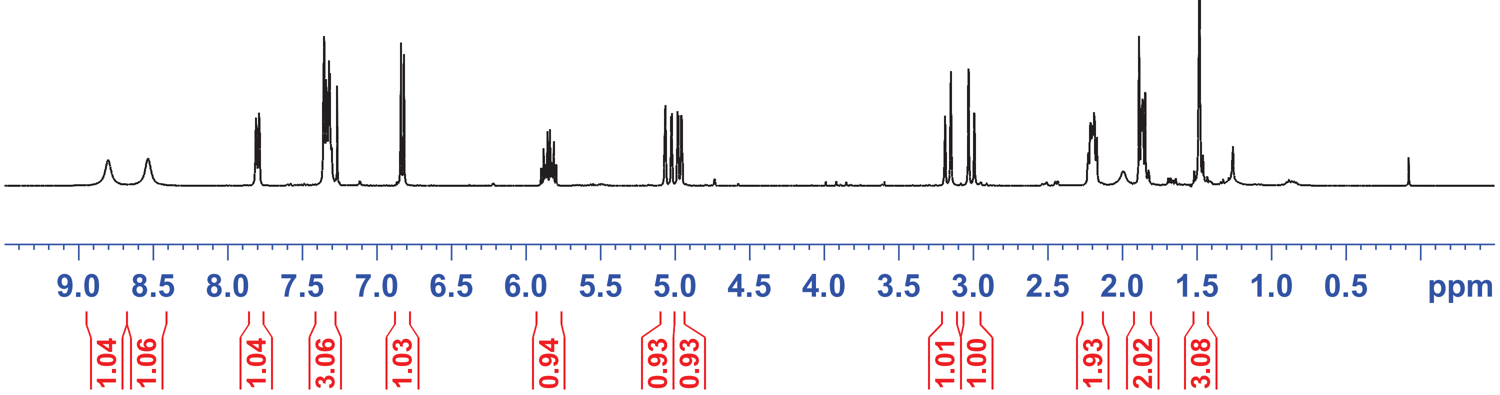

Current Data Parameters
NAME CDS-IX-37B
EXPNO 10
PROCNO 1

F2 - Acquisition Parameters
Date_ 20150918
Time 11.59
INSTRUM spect
PROBHD 5 mm PABBO BB-
PULPROG zg30
TD 74012
SOLVENT CDCl3
NS 16
DS 2
SWH 8223.685 Hz
FIDRES 0.111113 Hz
AQ 4.4999294 sec
RG 80.6
DW 60.800 usec
DE 16.87 usec
TE 297.4 K
D1 0.50000000 sec
TD0 1

======== CHANNEL f1 ========
SFO1 400.1924713 MHz
NUC1 1H
P1 10.00 usec
PLW1 23.03800011 W

F2 - Processing parameters
SI 131072
SF 400.1900081 MHz
WDW EM
SSB 0
LB 0.30 Hz
GB 0
PC 1.00

user Craig Smith
C13CPD1024.GLA CDCl3 /u craigsm 37

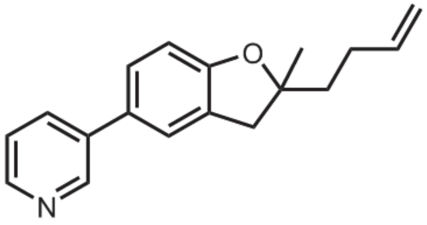

3f

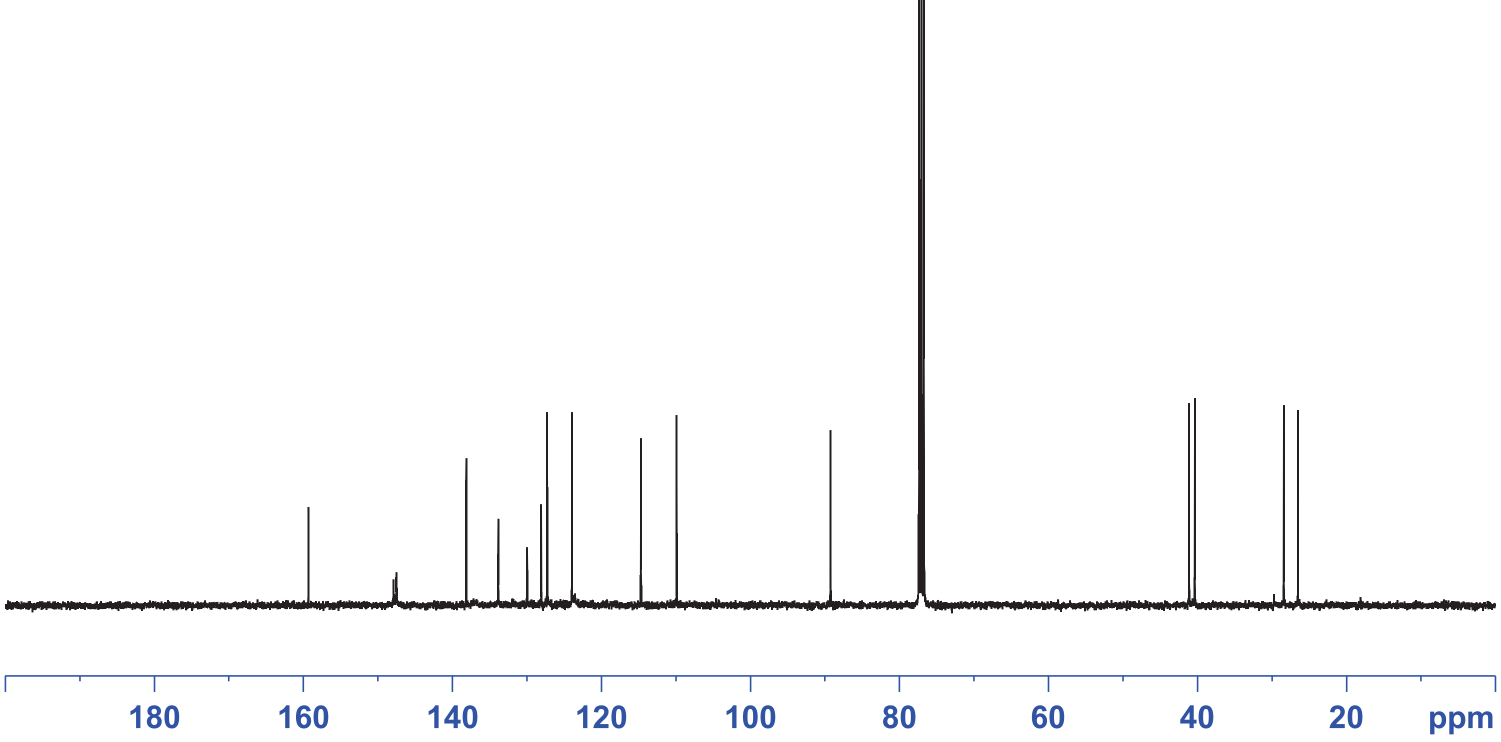

Current Data Parameters
NAME CDS-IX-37B
EXPNO 36
PROCNO 1

F2 - Acquisition Parameters
Date_ 20151030
Time 21.04
INSTRUM spect
PROBHD 5 mm PABBO BB-
PULPROG zgpg30
TD 65536
SOLVENT CDCl3
NS 1024
DS 4
SWH 24038.461 Hz
FIDRES 0.366798 Hz
AQ 1.3631488 sec
RG 2050
DW 20.800 usec
DE 9.78 usec
TE 298.0 K
D1 2.00000000 sec
D11 0.03000000 sec
TD0 1

======== CHANNEL f1 ========
SFO1 100.6379183 MHz
NUC1 13C
P1 9.00 usec
PLW1 51.32600021 W

======== CHANNEL f2 ========
SFO2 400.1916008 MHz
NUC2 1H
CPDPRG[2 waltz16
PCPD2 90.00 usec
PLW2 26.45100021 W
PLW12 0.26451001 W
PLW13 0.21425000 W

F2 - Processing parameters
SI 32768
SF 100.6278591 MHz
WDW EM
SSB 0
LB 1.00 Hz
GB 0
PC 1.40

user Craig Smith
proton.gla CDCl3 /u craigsm 40

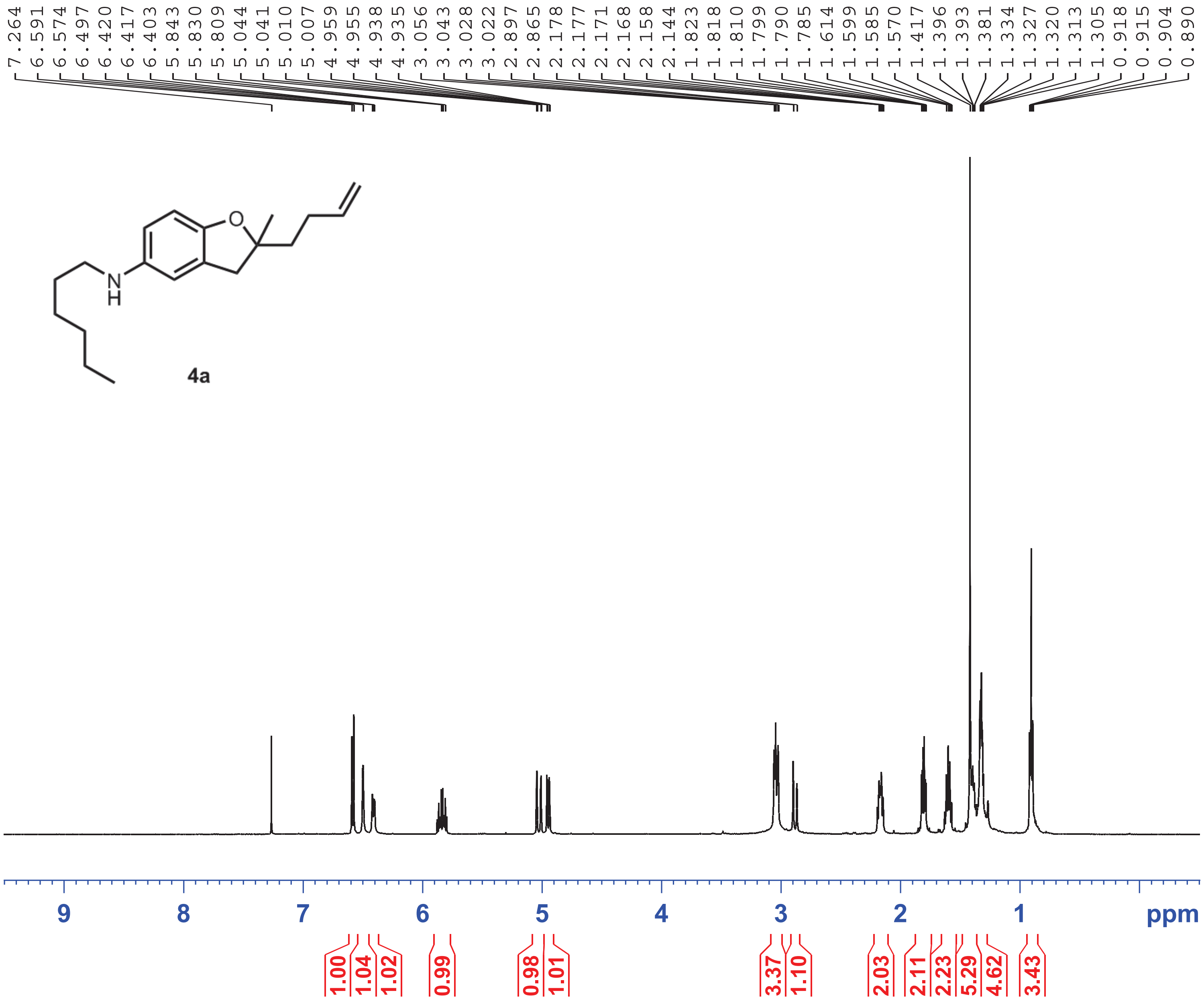

4a

Current Data Parameters
NAME CDS-VIII-36A
EXPNO 10
PROCNO 1

F2 - Acquisition Parameters
Date_ 20150401
Time 16.07
INSTRUM spect
PROBHD 5 mm PABBO BB-
PULPROG zg30
TD 74012
SOLVENT CDCl3
NS 16
DS 2
SWH 10273.973 Hz
FIDRES 0.138815 Hz
AQ 3.6019173 sec
RG 101
DW 48.667 usec
DE 7.02 usec
TE 295.7 K
D1 0.50000000 sec
TD0 1

======== CHANNEL f1 ========
SFO1 500.1930889 MHz
NUC1 1H
P1 11.00 usec
PLW1 18.32900047 W

F2 - Processing parameters
SI 131072
SF 500.1900096 MHz
WDW EM
SSB 0
LB 0.30 Hz
GB 0
PC 1.00

user Craig Smith
C13CPD256.GLA CDCl3 /u craigsm 40

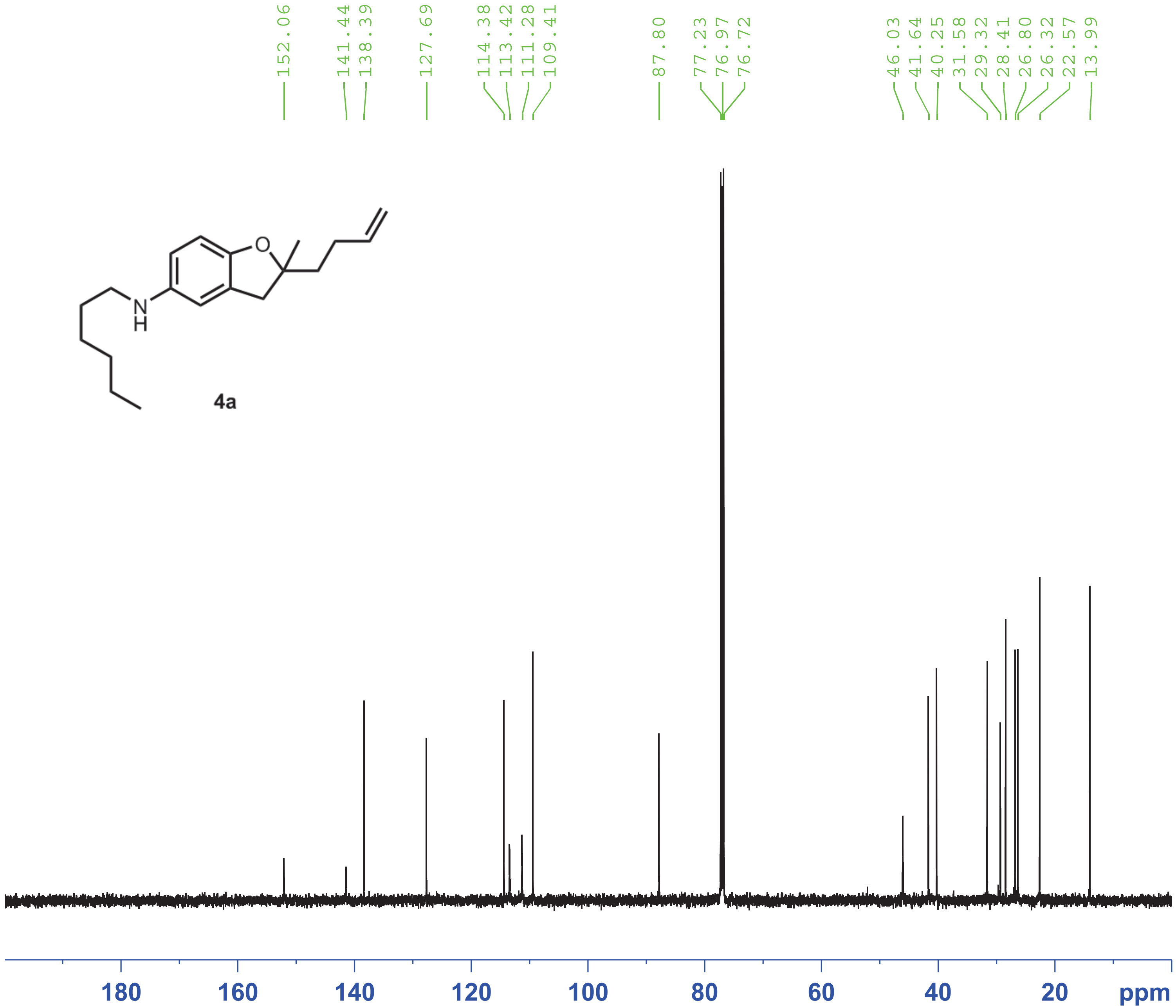

Current Data Parameters
NAME CDS-VIII-36A
EXPNO 22
PROCNO 1

F2 - Acquisition Parameters
Date_ 20150402
Time 22.29
INSTRUM spect
PROBHD 5 mm PABBO BB-
PULPROG zgpg30
TD 65536
SOLVENT CDCl3
NS 256
DS 4
SWH 30000.000 Hz
FIDRES 0.457764 Hz
AQ 1.0922667 sec
RG 2050
DW 16.667 usec
DE 8.01 usec
TE 296.7 K
D1 2.00000000 sec
D11 0.03000000 sec
TD0 1

======== CHANNEL f1 ========
SFO1 125.7854522 MHz
NUC1 13C
P1 7.50 usec
PLW1 147.41000366 W

======== CHANNEL f2 ========
SFO2 500.1920008 MHz
NUC2 1H
CPDPRG[2 waltz16
PCPD2 80.00 usec
PLW2 18.75499916 W
PLW12 0.38756001 W
PLW13 0.24804001 W

F2 - Processing parameters
SI 32768
SF 125.7728843 MHz
WDW EM
SSB 0
LB 1.00 Hz
GB 0
PC 1.40

user Craig Smith
proton.gla CDCl3 /u craigsm 36

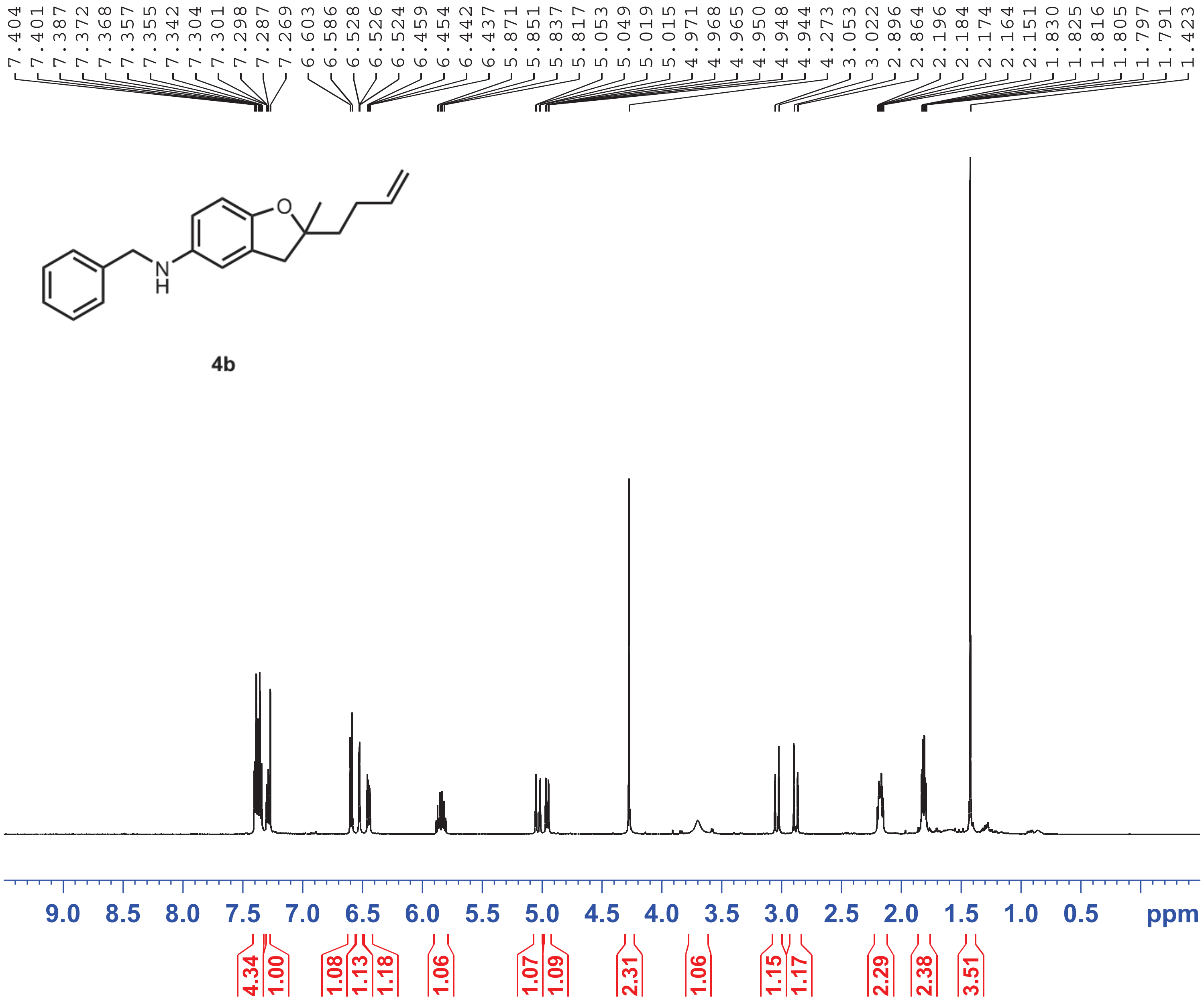

4b

Current Data Parameters
NAME CDS-X-44A
EXPNO 10
PROCNO 1

F2 - Acquisition Parameters
Date_ 20160412
Time 16.17
INSTRUM spect
PROBHD 5 mm PABBO BB-
PULPROG zg30
TD 74012
SOLVENT CDCl3
NS 16
DS 2
SWH 10273.973 Hz
FIDRES 0.138815 Hz
AQ 3.6019173 sec
RG 114
DW 48.667 usec
DE 12.48 usec
TE 297.2 K
D1 0.50000000 sec
TD0 1

======== CHANNEL f1 ========
SFO1 500.1930889 MHz
NUC1 1H
P1 14.00 usec
PLW1 18.32900047 W

F2 - Processing parameters
SI 131072
SF 500.1900072 MHz
WDW EM
SSB 0
LB 0.30 Hz
GB 0
PC 1.00

user Craig Smith
C13CPD1024.GLA CDCl3 /u craigsm 39

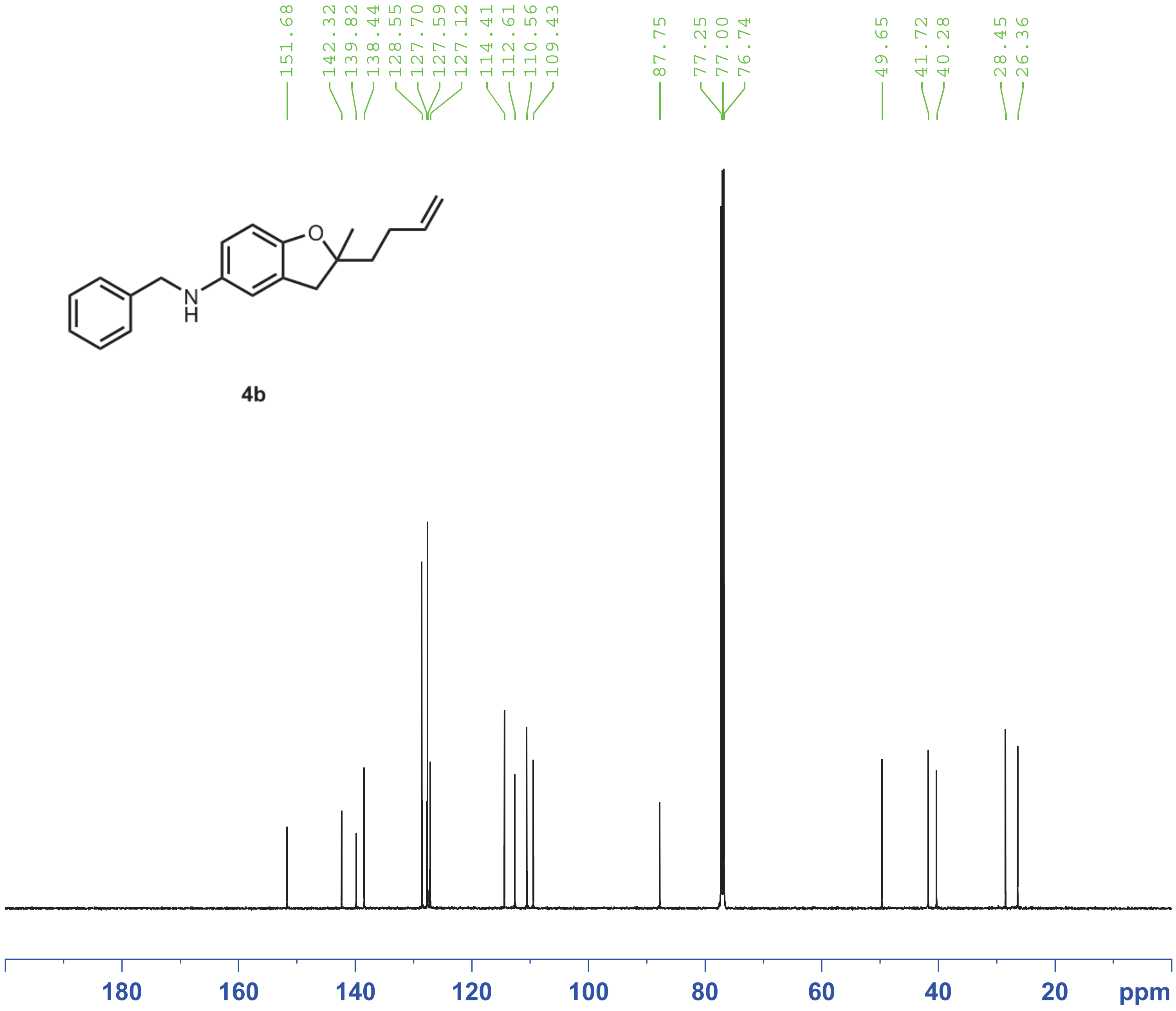

Current Data Parameters
NAME CDS-X-44A
EXPNO 31
PROCNO 1

F2 - Acquisition Parameters
Date_ 20160412
Time 21.34
INSTRUM spect
PROBHD 5 mm PABBO BB-
PULPROG zgpg30
TD 65536
SOLVENT CDCl3
NS 5000
DS 4
SWH 30000.000 Hz
FIDRES 0.457764 Hz
AQ 1.0922667 sec
RG 2050
DW 16.667 usec
DE 7.76 usec
TE 297.2 K
D1 2.00000000 sec
D11 0.03000000 sec
TD0 1

======== CHANNEL f1 ========
SFO1 125.7854522 MHz
NUC1 13C
P1 8.70 usec
PLW1 120.00000000 W

======== CHANNEL f2 ========
SFO2 500.1920008 MHz
NUC2 1H
CPDPRG[2 waltz16
PCPD2 80.00 usec
PLW2 18.75499916 W
PLW12 0.57437003 W
PLW13 0.36759999 W

F2 - Processing parameters
SI 32768
SF 125.7728808 MHz
WDW EM
SSB 0
LB 1.00 Hz
GB 0
PC 1.40

user Craig Smith
PROTON.GLA CDCl3 /u craigsm 24

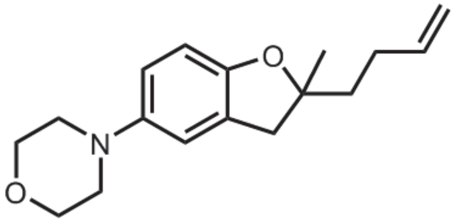

4c

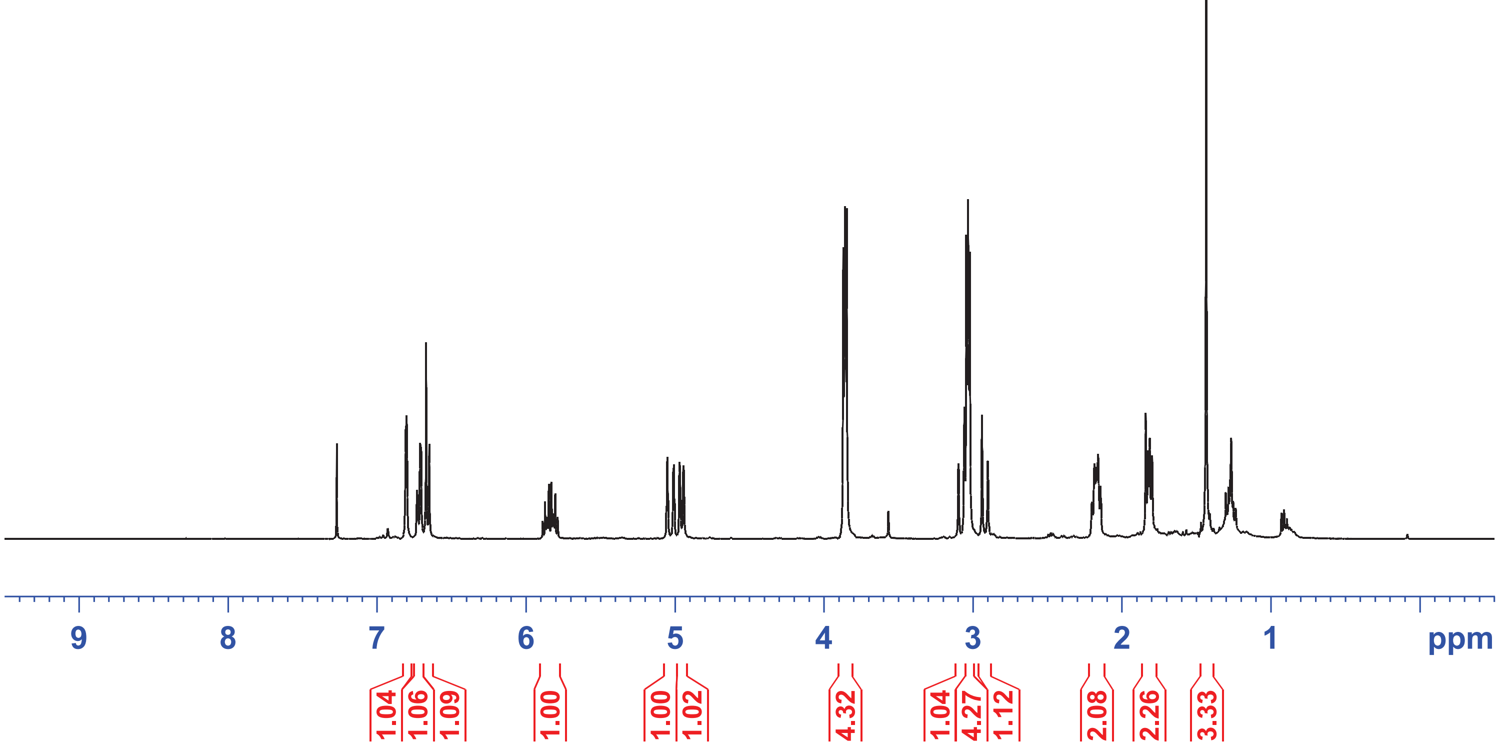

Current Data Parameters

NAME	CDS-VIII-45A_2
EXPNO	10
PROCNO	1

F2 - Acquisition Parameters

Date_	20150416
Time	11.14
INSTRUM	spect
PROBHD	5 mm PABBO BB-
PULPROG	zg30
TD	74012
SOLVENT	CDCl3
NS	16
DS	2
SWH	8223.685 Hz
FIDRES	0.111113 Hz
AQ	4.4999294 sec
RG	80.6
DW	60.800 usec
DE	16.87 usec
TE	295.8 K
D1	0.50000000 sec
TD0	1

======== CHANNEL f1 ========

SFO1	400.1924713 MHz
NUC1	1H
P1	10.00 usec
PLW1	23.03800011 W

F2 - Processing parameters

SI	131072
SF	400.1900064 MHz
WDW	EM
SSB	0
LB	0.30 Hz
GB	0
PC	1.00

user Craig Smith
C13CPD1024.GLA CDCl3 /u craigsm 24

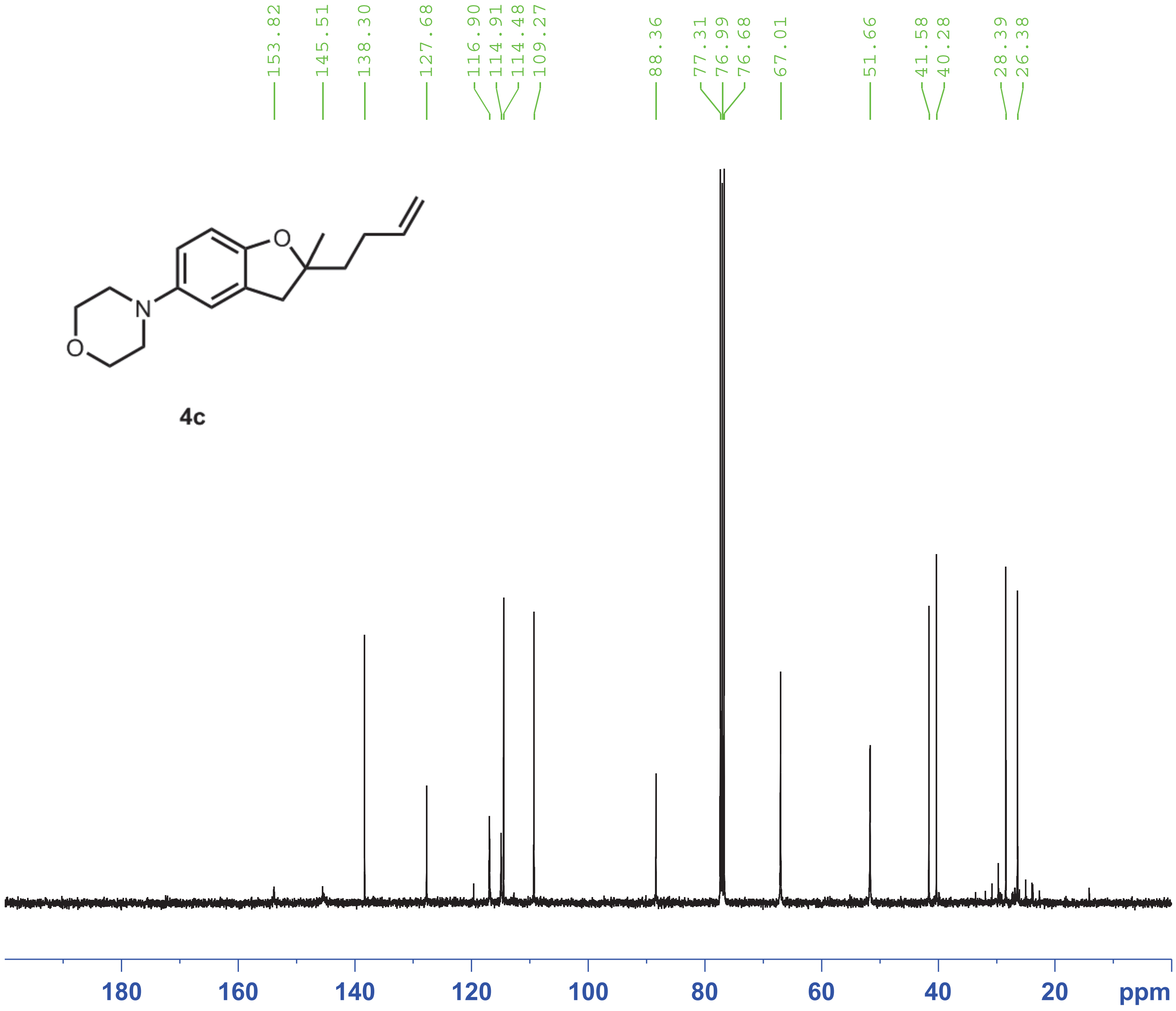

4c

```
Current Data Parameters
NAME          CDS-VIII-45A
EXPNO                   16
PROCNO                   1

F2 - Acquisition Parameters
Date_             20150416
Time                 23.08
INSTRUM              spect
PROBHD     5 mm PABBO BB-
PULPROG            zgpg30
TD                   65536
SOLVENT              CDCl3
NS                    1024
DS                       4
SWH             24038.461 Hz
FIDRES           0.366798 Hz
AQ              1.3631488 sec
RG                    2050
DW                  20.800 usec
DE                    9.78 usec
TE                   297.2 K
D1             2.00000000 sec
D11            0.03000000 sec
TD0                      1

======== CHANNEL f1 ========
SFO1          100.6379183 MHz
NUC1                  13C
P1                   9.00 usec
PLW1          51.32600021 W

======== CHANNEL f2 ========
SFO2          400.1916008 MHz
NUC2                    1H
CPDPRG[2           waltz16
PCPD2               90.00 usec
PLW2          26.45100021 W
PLW12          0.26451001 W
PLW13          0.21425000 W

F2 - Processing parameters
SI                   32768
SF            100.6278597 MHz
WDW                     EM
SSB        0
LB                    1.00 Hz
GB         0
PC                    1.40
```

user Craig Smith
PROTON.GLA CDCl3 /u craigsm 29

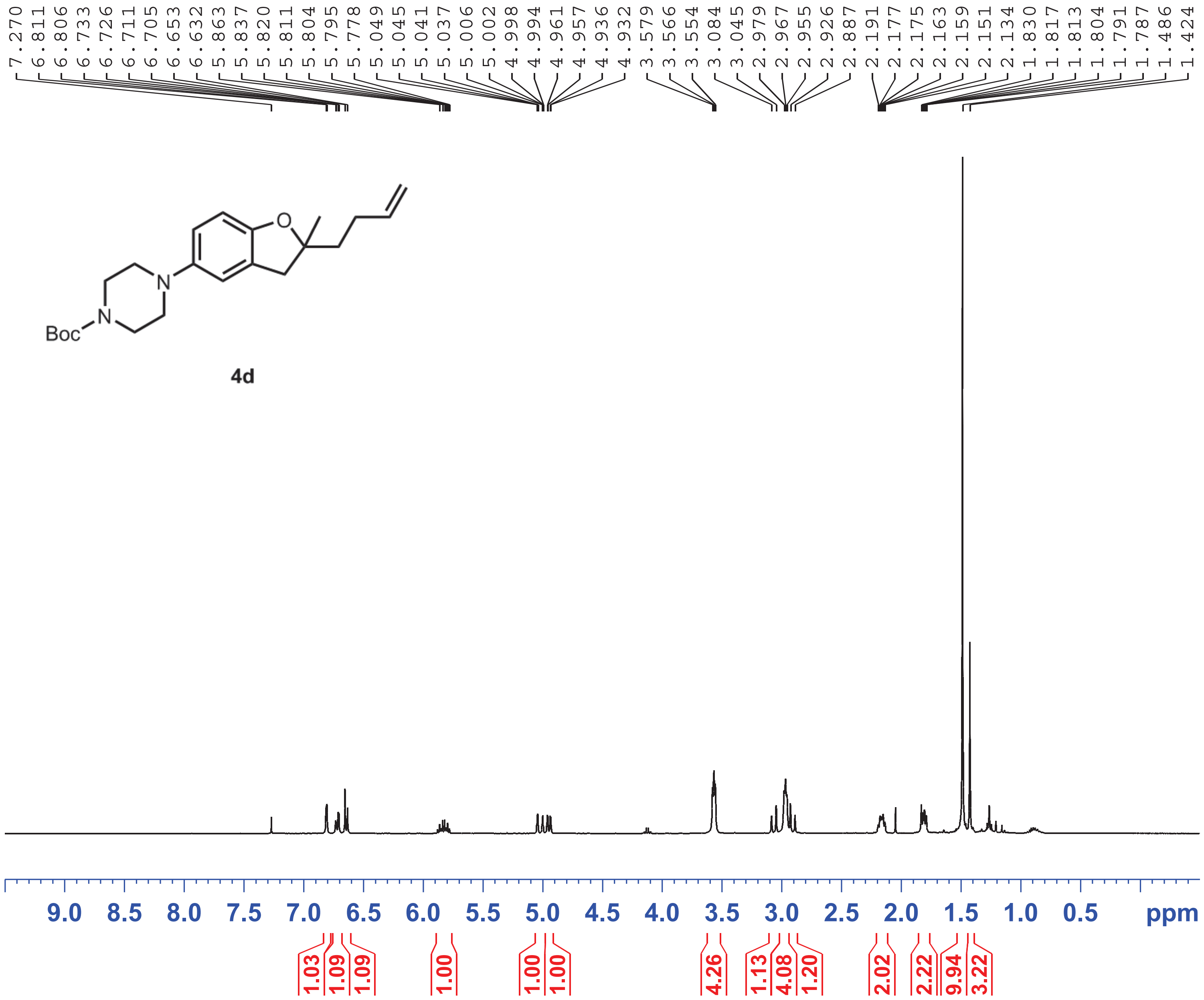

4d

Current Data Parameters
NAME CDS-VIII-58A
EXPNO 10
PROCNO 1

F2 - Acquisition Parameters
Date_ 20150508
Time 14.02
INSTRUM spect
PROBHD 5 mm PABBO BB-
PULPROG zg30
TD 74012
SOLVENT CDCl3
NS 16
DS 2
SWH 8223.685 Hz
FIDRES 0.111113 Hz
AQ 4.4999294 sec
RG 50.8
DW 60.800 usec
DE 16.87 usec
TE 297.3 K
D1 0.50000000 sec
TD0 1

======== CHANNEL f1 ========
SFO1 400.1924713 MHz
NUC1 1H
P1 10.00 usec
PLW1 23.03800011 W

F2 - Processing parameters
SI 131072
SF 400.1900060 MHz
WDW EM
SSB 0
LB 0.30 Hz
GB 0
PC 1.00

user Craig Smith
C13CPD1024.GLA CDCl3 /u craigsm 29

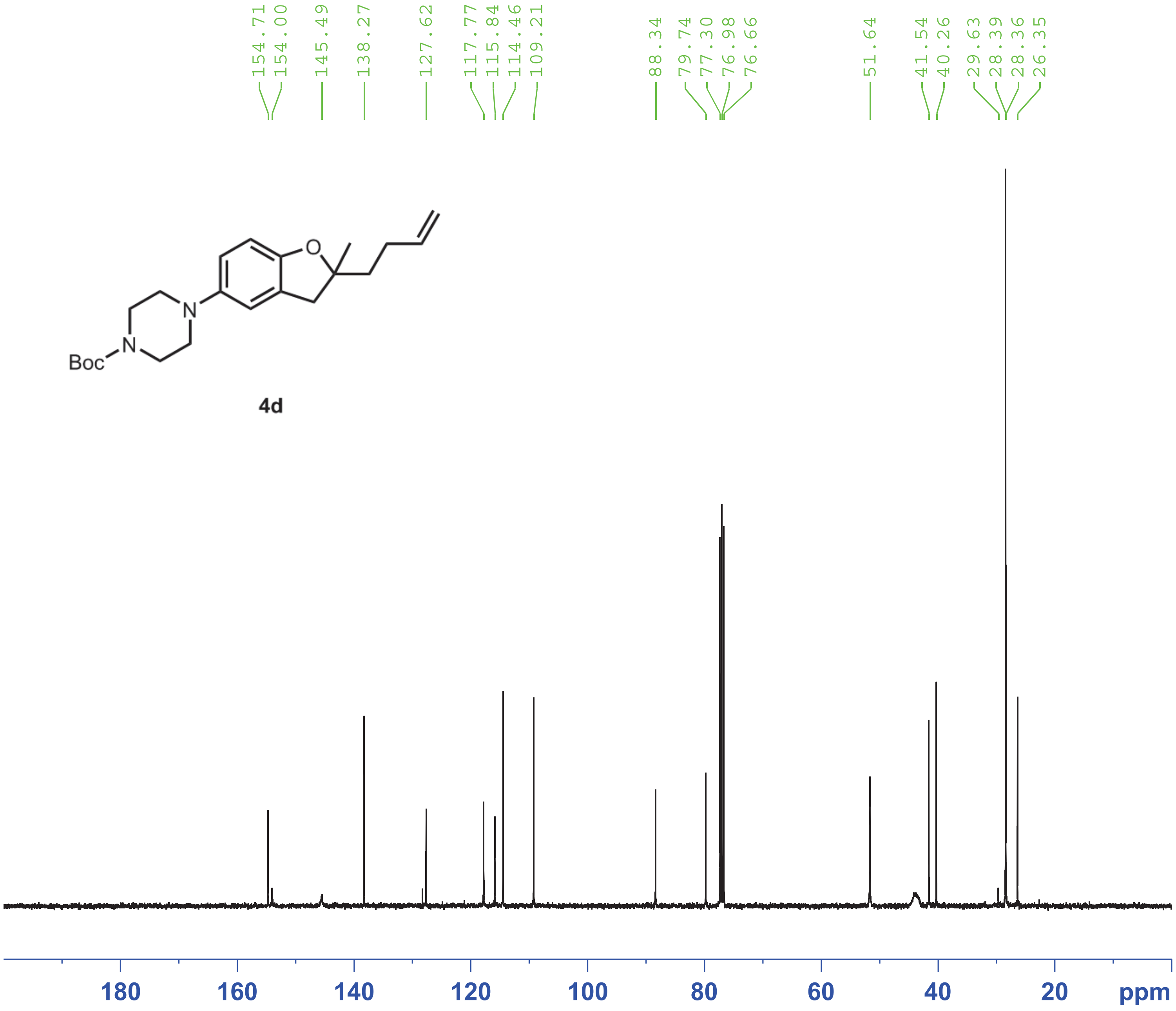

Current Data Parameters
NAME CDS-VIII-58A
EXPNO 26
PROCNO 1

F2 - Acquisition Parameters
Date_ 20150511
Time 22.32
INSTRUM spect
PROBHD 5 mm PABBO BB-
PULPROG zgpg30
TD 65536
SOLVENT CDCl3
NS 1024
DS 4
SWH 24038.461 Hz
FIDRES 0.366798 Hz
AQ 1.3631488 sec
RG 2050
DW 20.800 usec
DE 9.78 usec
TE 298.6 K
D1 2.00000000 sec
D11 0.03000000 sec
TD0 1

======== CHANNEL f1 ========
SFO1 100.6379183 MHz
NUC1 13C
P1 9.00 usec
PLW1 51.32600021 W

======== CHANNEL f2 ========
SFO2 400.1916008 MHz
NUC2 1H
CPDPRG[2 waltz16
PCPD2 90.00 usec
PLW2 26.45100021 W
PLW12 0.26451001 W
PLW13 0.21425000 W

F2 - Processing parameters
SI 32768
SF 100.6278613 MHz
WDW EM
SSB 0
LB 1.00 Hz
GB 0
PC 1.40

user Craig Smith
proton.gla CDCl3 /u craigsm 55

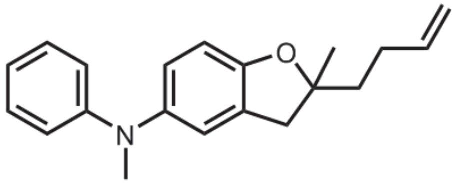

4e

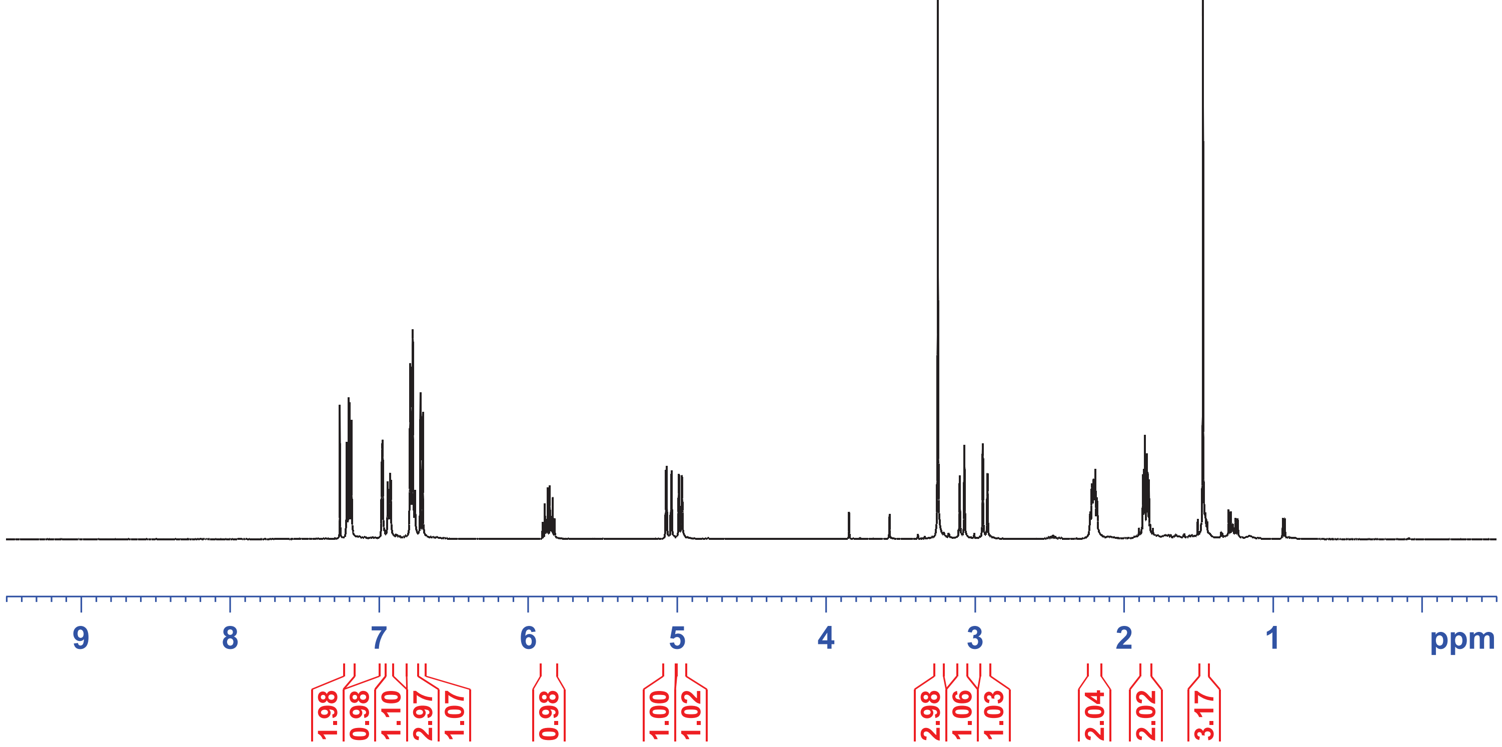


```
Current Data Parameters
NAME            CDS-VIII-56A
EXPNO                     30
PROCNO                     1

F2 - Acquisition Parameters
Date_               20150506
Time                   22.02
INSTRUM                spect
PROBHD     5 mm PABBO BB-
PULPROG                 zg30
TD                     74012
SOLVENT                CDCl3
NS                        16
DS                         2
SWH               10273.973 Hz
FIDRES             0.138815 Hz
AQ                3.6019173 sec
RG                       128
DW                    48.667 usec
DE                     12.48 usec
TE                     296.1 K
D1                0.50000000 sec
TD0                        1

======== CHANNEL f1 ========
SFO1           500.1930889 MHz
NUC1                      1H
P1                     14.00 usec
PLW1             18.32900047 W

F2 - Processing parameters
SI                    131072
SF             500.1900107 MHz
WDW                       EM
SSB      0
LB                      0.30 Hz
GB       0
PC                      1.00
```

user Craig Smith
C13CPD1024.GLA CDCl3 /u craigsm 55

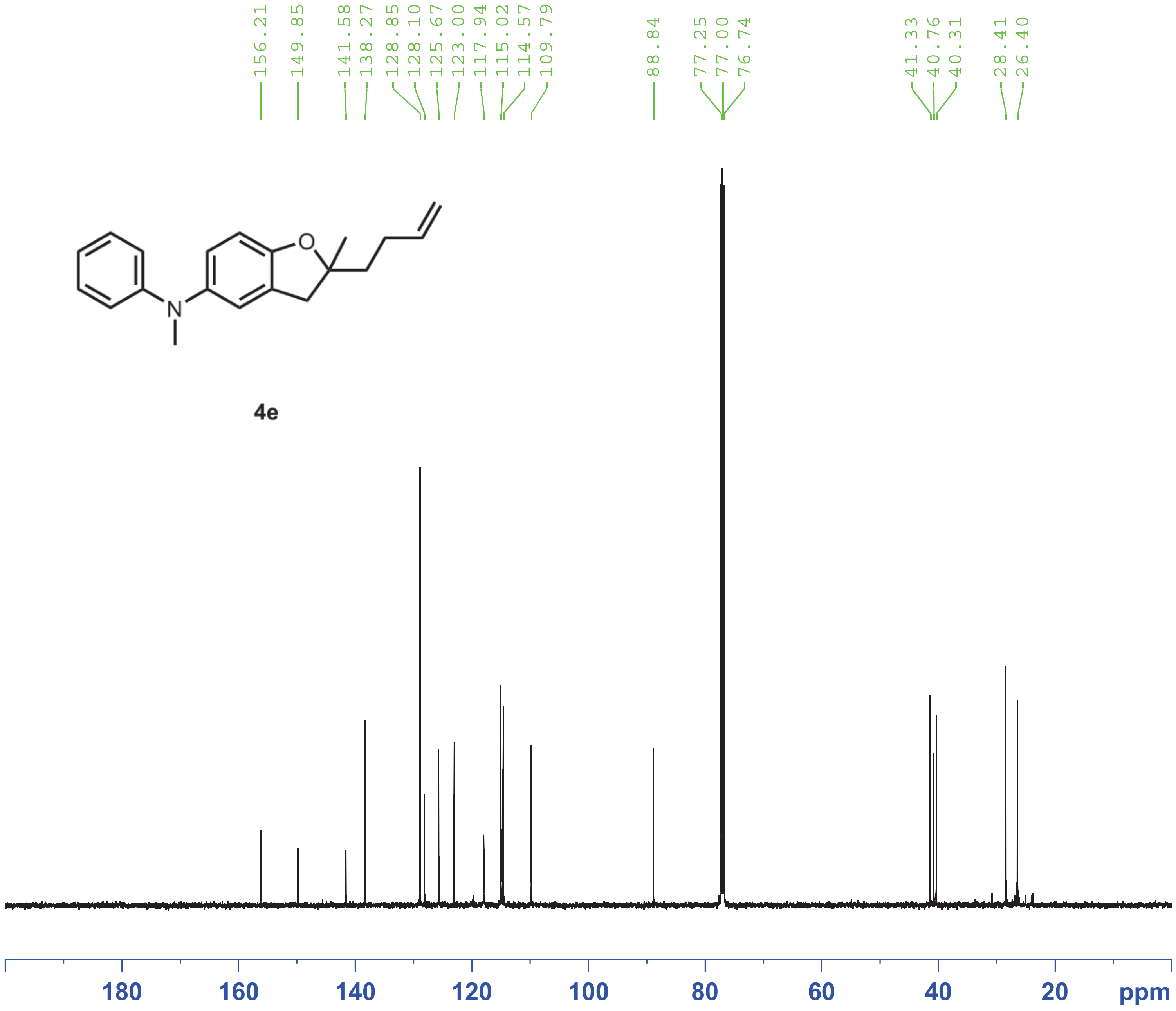

4e

```
Current Data Parameters
NAME          CDS-VIII-56A
EXPNO                   31
PROCNO                   1

F2 - Acquisition Parameters
Date_             20150506
Time                 22.58
INSTRUM              spect
PROBHD     5 mm PABBO BB-
PULPROG            zgpg30
TD                   65536
SOLVENT              CDCl3
NS                    1024
DS                       4
SWH              30000.000 Hz
FIDRES            0.457764 Hz
AQ               1.0922667 sec
RG                    2050
DW                  16.667 usec
DE                    7.90 usec
TE                   297.3 K
D1              2.00000000 sec
D11             0.03000000 sec
TD0                      1

======== CHANNEL f1 ========
SFO1           125.7854522 MHz
NUC1                   13C
P1                    8.00 usec
PLW1          147.41000366 W

======== CHANNEL f2 ========
SFO2           500.1920008 MHz
NUC2                    1H
CPDPRG[2           waltz16
PCPD2                80.00 usec
PLW2           18.75499916 W
PLW12           0.57437003 W
PLW13           0.36759999 W

F2 - Processing parameters
SI                   32768
SF             125.7728810 MHz
WDW                     EM
SSB        0
LB                    1.00 Hz
GB         0
PC                    1.40
```

user Craig Smith
PROTON.GLA CDCl3 /u craigsm 33

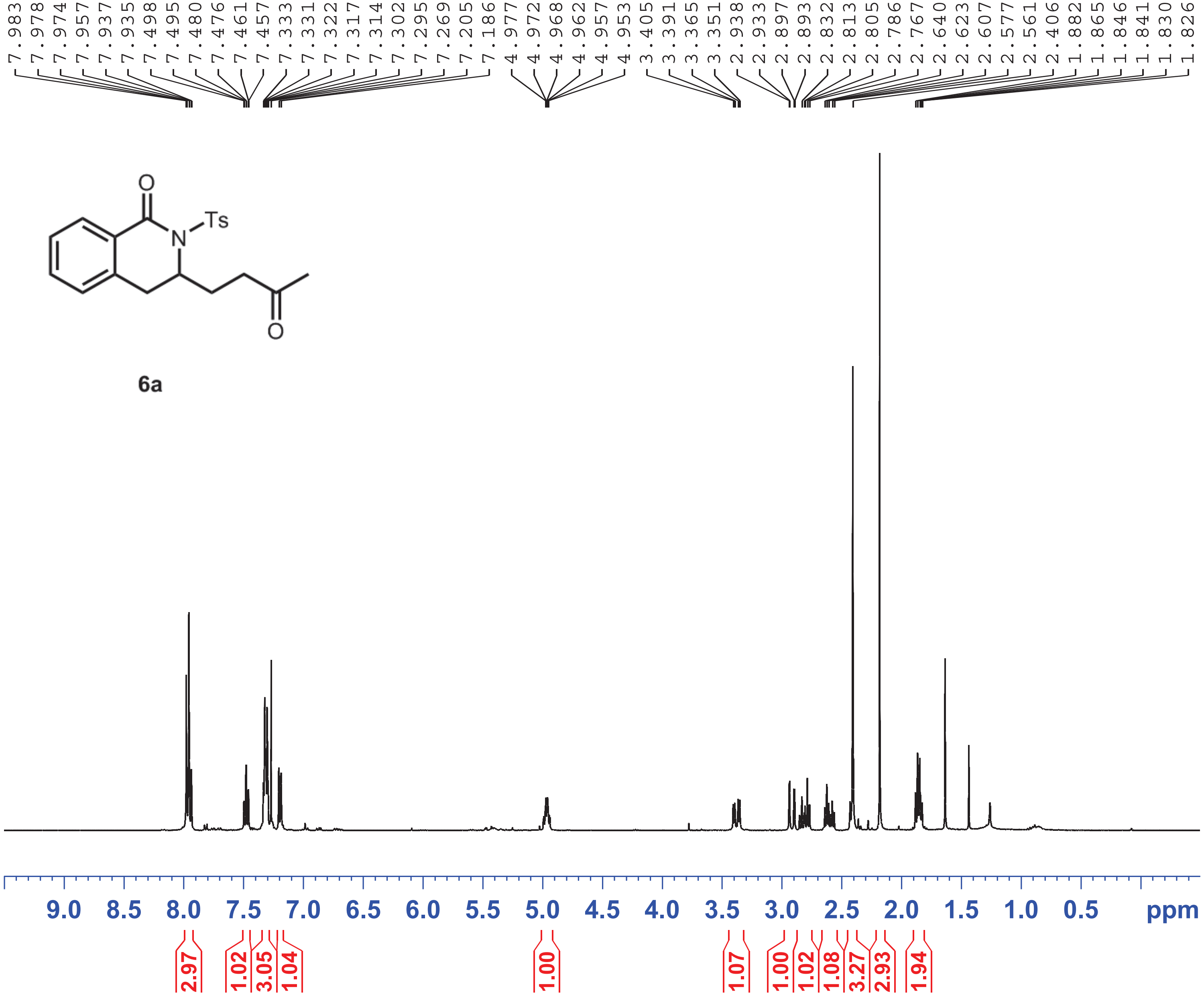

6a

Current Data Parameters
NAME CDS-IX-93A
EXPNO 10
PROCNO 1

F2 - Acquisition Parameters
Date_ 20151215
Time 15.59
INSTRUM spect
PROBHD 5 mm PABBO BB-
PULPROG zg30
TD 74012
SOLVENT CDCl3
NS 16
DS 2
SWH 8223.685 Hz
FIDRES 0.111113 Hz
AQ 4.4999294 sec
RG 181
DW 60.800 usec
DE 16.87 usec
TE 294.9 K
D1 0.50000000 sec
TD0 1

======== CHANNEL f1 ========
SFO1 400.1924713 MHz
NUC1 1H
P1 10.00 usec
PLW1 23.03800011 W

F2 - Processing parameters
SI 131072
SF 400.1900066 MHz
WDW EM
SSB 0
LB 0.30 Hz
GB 0
PC 1.00

user Craig Smith
C13CPD1024.GLA CDCl3 /u craigsm 33

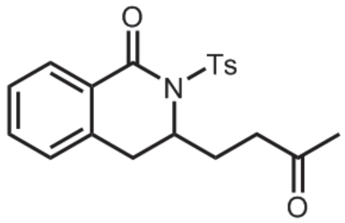

6a

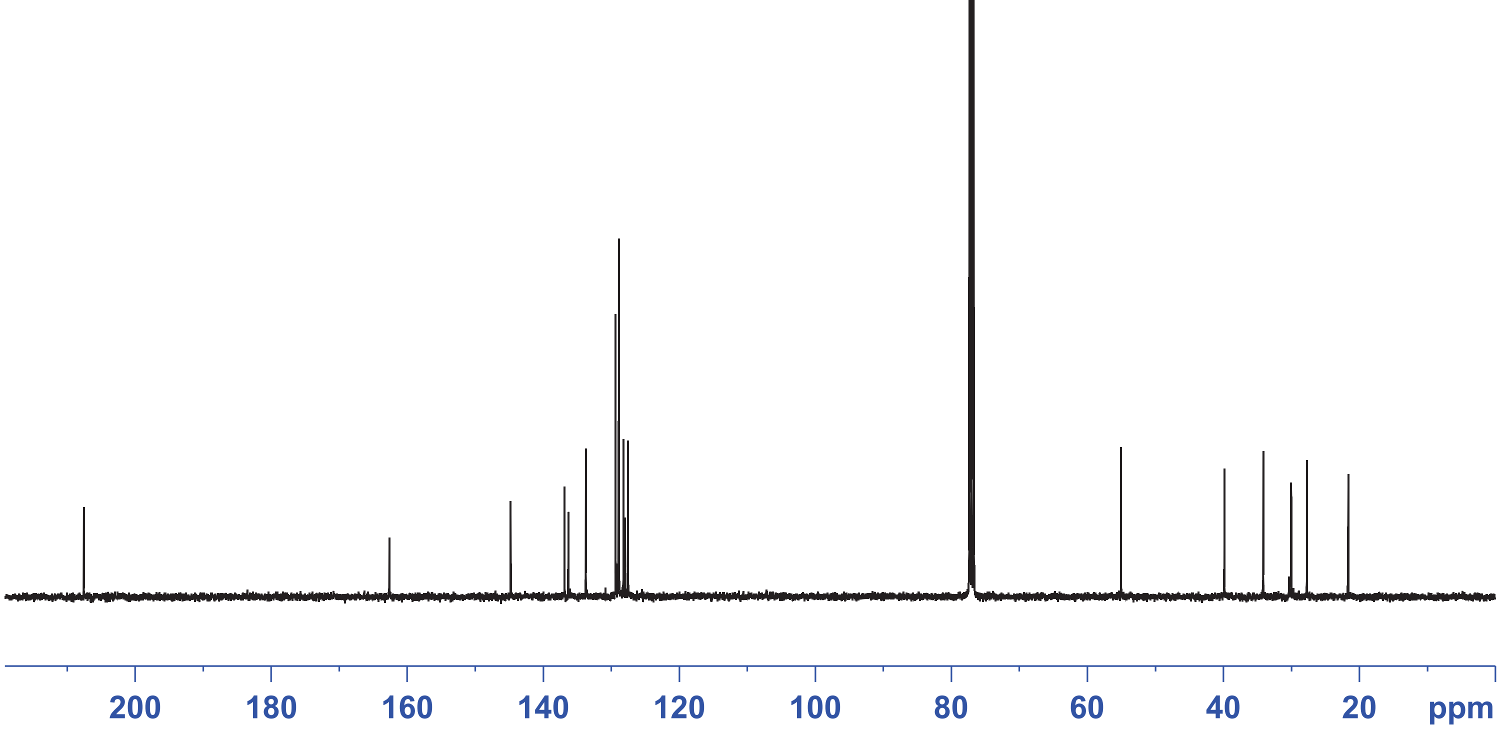

Current Data Parameters
NAME CDS-IX-93A
EXPNO 25
PROCNO 1

F2 - Acquisition Parameters
Date_ 20151217
Time 21.58
INSTRUM spect
PROBHD 5 mm PABBO BB-
PULPROG zgpg30
TD 65536
SOLVENT CDCl3
NS 1024
DS 4
SWH 24038.461 Hz
FIDRES 0.366798 Hz
AQ 1.3631488 sec
RG 2050
DW 20.800 usec
DE 9.78 usec
TE 299.2 K
D1 2.00000000 sec
D11 0.03000000 sec
TD0 1

======== CHANNEL f1 ========
SFO1 100.6379183 MHz
NUC1 13C
P1 9.00 usec
PLW1 51.32600021 W

======== CHANNEL f2 ========
SFO2 400.1916008 MHz
NUC2 1H
CPDPRG[2 waltz16
PCPD2 90.00 usec
PLW2 26.45100021 W
PLW12 0.26451001 W
PLW13 0.21425000 W

F2 - Processing parameters
SI 32768
SF 100.6278603 MHz
WDW EM
SSB 0
LB 1.00 Hz
GB 0
PC 1.40

user Craig Smith
PROTON.GLA CDCl3 /u craigsm 55

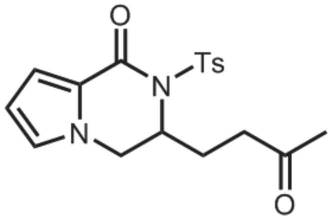

6b

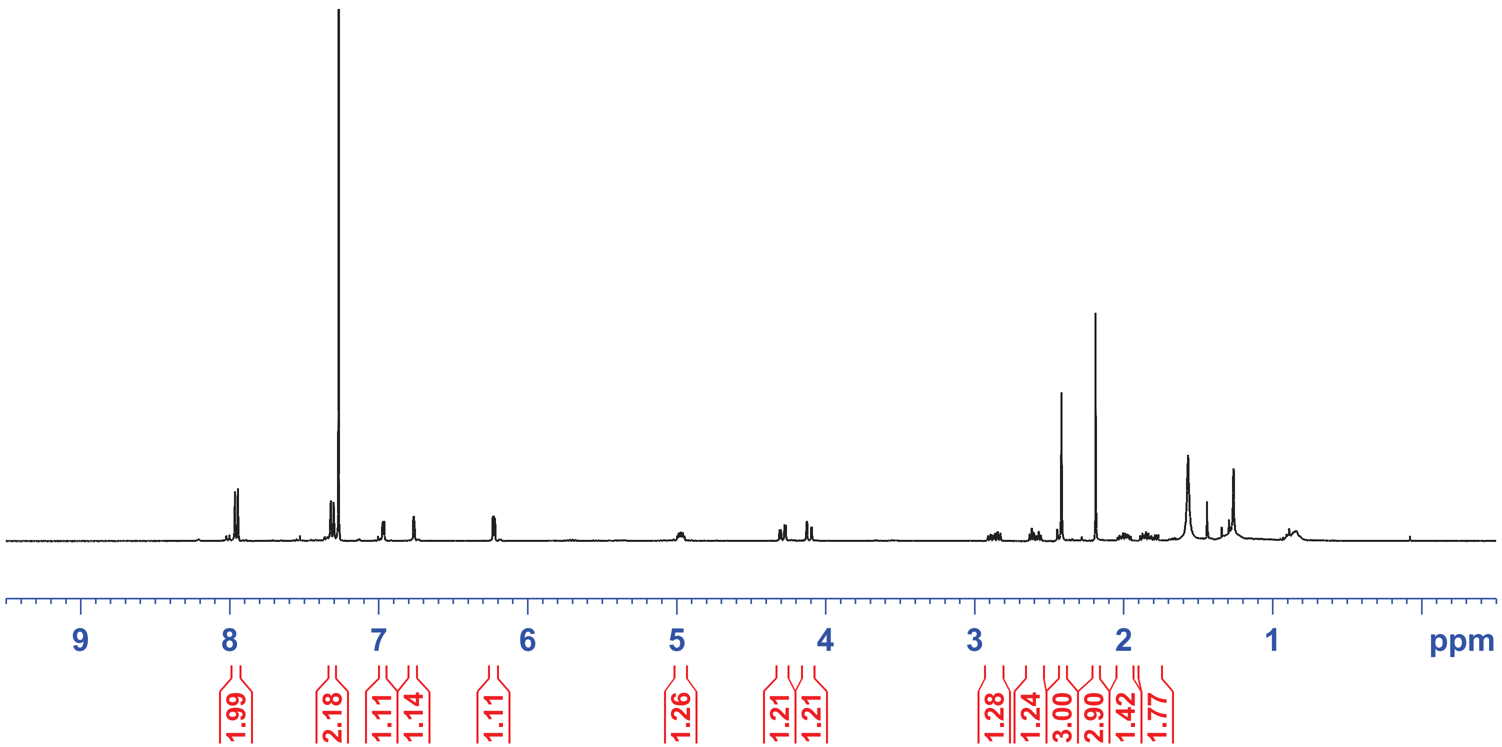

Current Data Parameters
NAME CDS-X-46D
EXPNO 20
PROCNO 1

F2 - Acquisition Parameters
Date_ 20160422
Time 6.32
INSTRUM spect
PROBHD 5 mm PABBO BB-
PULPROG zg30
TD 74012
SOLVENT CDCl3
NS 200
DS 2
SWH 8223.685 Hz
FIDRES 0.111113 Hz
AQ 4.4999294 sec
RG 406
DW 60.800 usec
DE 16.87 usec
TE 296.7 K
D1 0.50000000 sec
TD0 1

======== CHANNEL f1 ========
SFO1 400.1924713 MHz
NUC1 1H
P1 10.00 usec
PLW1 23.03800011 W

F2 - Processing parameters
SI 131072
SF 400.1900058 MHz
WDW EM
SSB 0
LB 0.30 Hz
GB 0
PC 1.00

user Craig Smith
C13CPD1024.GLA CDCl3 /u craigsm 55

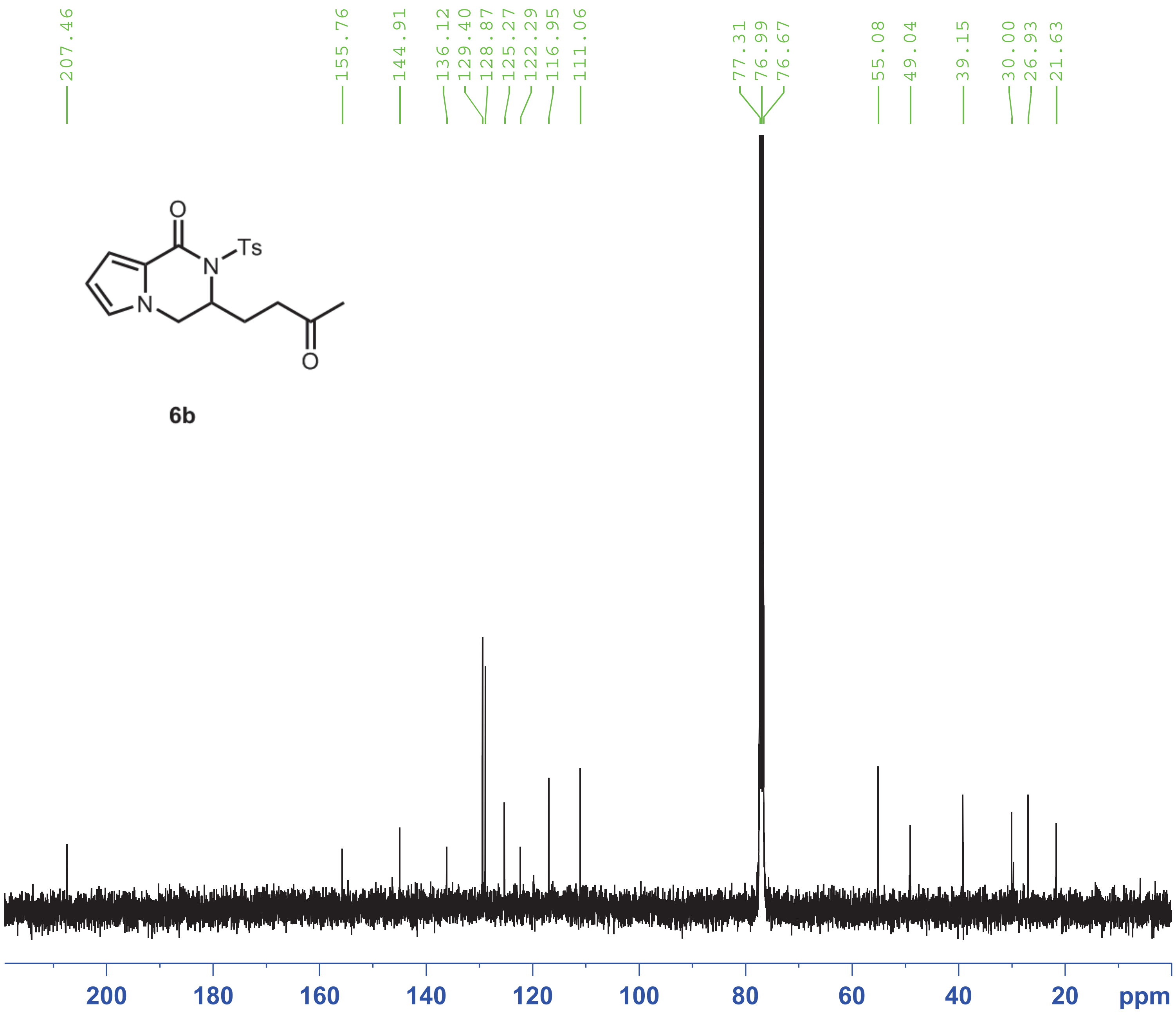

Current Data Parameters
NAME CDS-X-46D
EXPNO 22
PROCNO 1

F2 - Acquisition Parameters
Date_ 20160423
Time 1.47
INSTRUM spect
PROBHD 5 mm PABBO BB-
PULPROG zgpg30
TD 65536
SOLVENT CDCl3
NS 6000
DS 4
SWH 24038.461 Hz
FIDRES 0.366798 Hz
AQ 1.3631488 sec
RG 2050
DW 20.800 usec
DE 9.78 usec
TE 297.2 K
D1 2.00000000 sec
D11 0.03000000 sec
TD0 1

======== CHANNEL f1 ========
SFO1 100.6379183 MHz
NUC1 13C
P1 9.00 usec
PLW1 51.32600021 W

======== CHANNEL f2 ========
SFO2 400.1916008 MHz
NUC2 1H
CPDPRG[2 waltz16
PCPD2 90.00 usec
PLW2 26.45100021 W
PLW12 0.26451001 W
PLW13 0.21425000 W

F2 - Processing parameters
SI 32768
SF 100.6278576 MHz
WDW EM
SSB 0
LB 1.00 Hz
GB 0
PC 1.40

user Craig Smith
PROTON.GLA CDCl3 /u craigsm 44

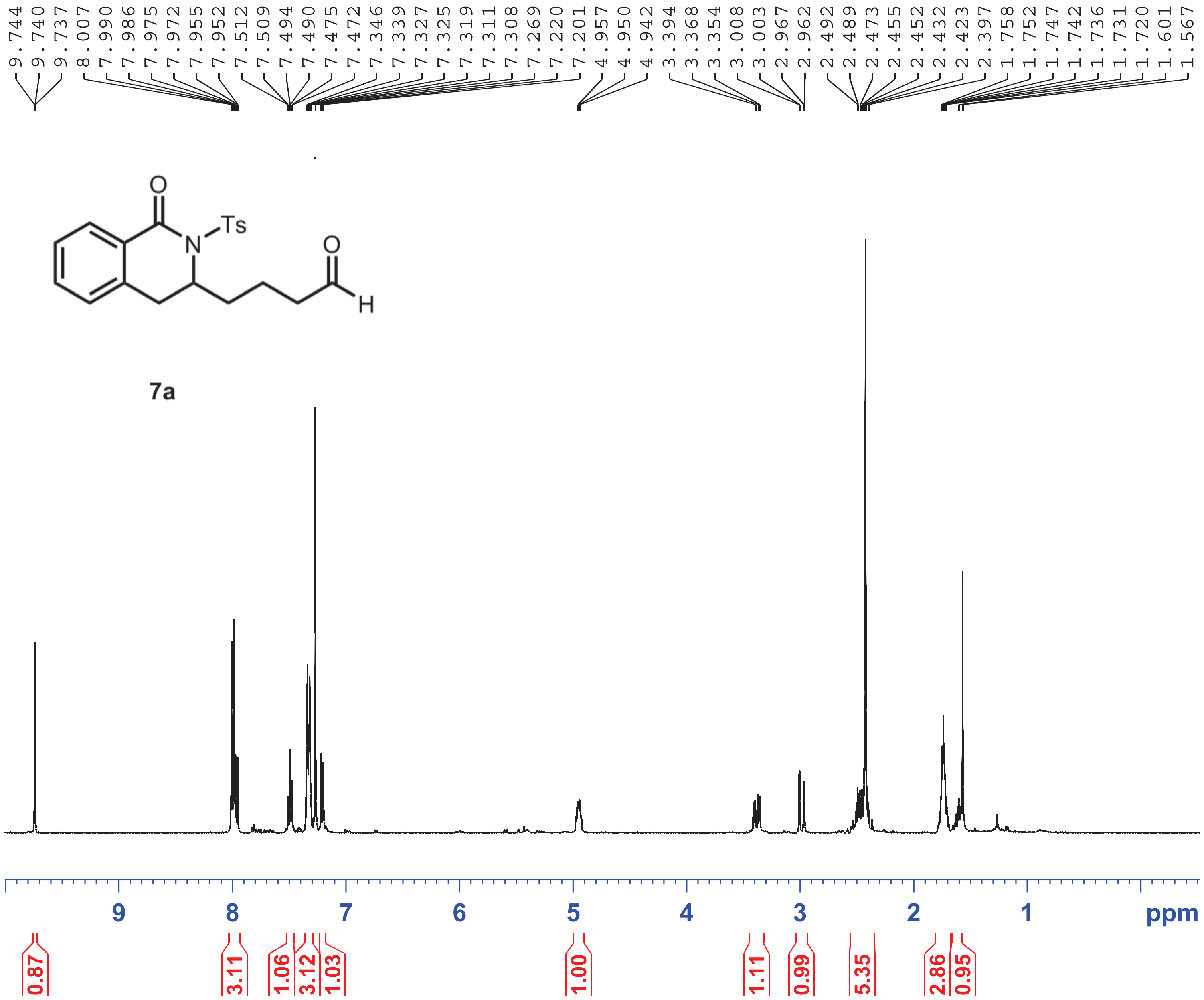

7a

Current Data Parameters
NAME CDS-X-28C
EXPNO 10
PROCNO 1

F2 - Acquisition Parameters
Date_ 20160314
Time 12.16
INSTRUM spect
PROBHD 5 mm PABBO BB-
PULPROG zg30
TD 74012
SOLVENT CDCl3
NS 16
DS 2
SWH 8223.685 Hz
FIDRES 0.111113 Hz
AQ 4.4999294 sec
RG 322
DW 60.800 usec
DE 16.87 usec
TE 297.2 K
D1 0.50000000 sec
TD0 1

======== CHANNEL f1 ========
SFO1 400.1924713 MHz
NUC1 1H
P1 10.00 usec
PLW1 23.03800011 W

F2 - Processing parameters
SI 131072
SF 400.1900062 MHz
WDW EM
SSB 0
LB 0.30 Hz
GB 0
PC 1.00

user Craig Smith
C13CPD1024.GLA CDCl3 /u craigsm 12

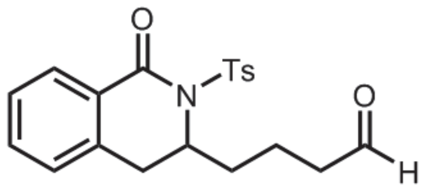

7a

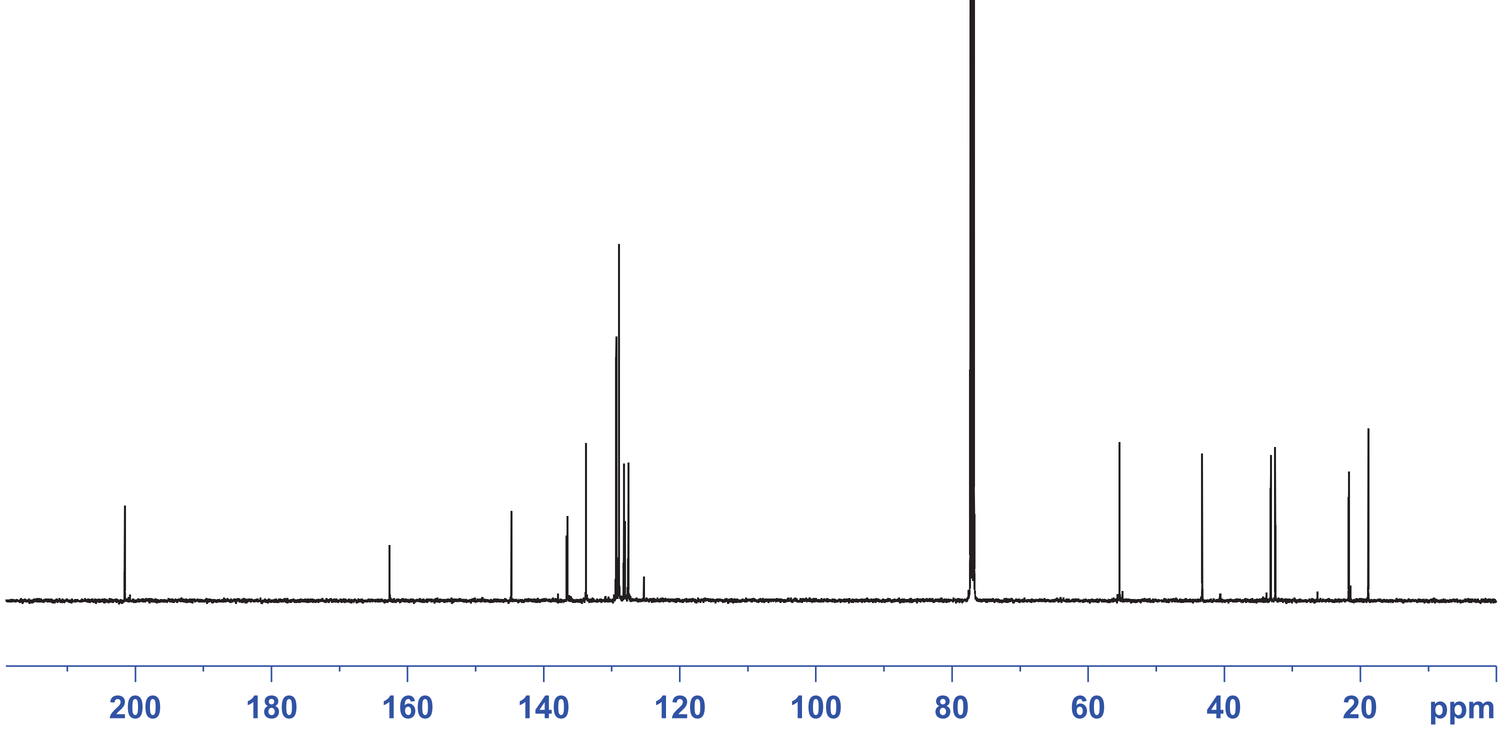


```
Current Data Parameters
NAME              CDS-X-45D
EXPNO                    21
PROCNO                    1

F2 - Acquisition Parameters
Date_              20160413
Time                  21.30
INSTRUM               spect
PROBHD     5 mm PABBO BB-
PULPROG             zgpg30
TD                    65536
SOLVENT               CDCl3
NS                     5000
DS                        4
SWH            30000.000 Hz
FIDRES          0.457764 Hz
AQ             1.0922667 sec
RG                     2050
DW                16.667 usec
DE                  7.76 usec
TE                  297.2 K
D1            2.00000000 sec
D11           0.03000000 sec
TD0                       1

======== CHANNEL f1 ========
SFO1         125.7854522 MHz
NUC1                    13C
P1                  8.70 usec
PLW1         120.00000000 W

======== CHANNEL f2 ========
SFO2         500.1920008 MHz
NUC2                     1H
CPDPRG[2            waltz16
PCPD2              80.00 usec
PLW2         18.75499916 W
PLW12         0.57437003 W
PLW13         0.36759999 W

F2 - Processing parameters
SI                    32768
SF           125.7728805 MHz
WDW                      EM
SSB        0
LB                  1.00 Hz
GB         0
PC                     1.40
```
